# Immune boosting by B.1.1.529 **(**Omicron) depends on previous SARS-CoV-2 exposure

**DOI:** 10.1126/science.abq1841

**Published:** 2022-06-14

**Authors:** Catherine J. Reynolds, Corinna Pade, Joseph M. Gibbons, Ashley D. Otter, Kai-Min Lin, Diana Muñoz Sandoval, Franziska P. Pieper, David K. Butler, Siyi Liu, George Joy, Nasim Forooghi, Thomas A. Treibel, Charlotte Manisty, James C. Moon, COVIDsortium Investigators, COVIDsortium Immune Correlates Network, Amanda Semper, Tim Brooks, Áine McKnight, Daniel M. Altmann, Rosemary J. Boyton, Hakam Abbass, Aderonke Abiodun, Mashael Alfarih, Zoe Alldis, Daniel M. Altmann, Oliver E. Amin, Mervyn Andiapen, Jessica Artico, João B. Augusto, Georgina L. Baca, Sasha N. L. Bailey, Anish N. Bhuva, Alex Boulter, Ruth Bowles, Rosemary J. Boyton, Olivia V. Bracken, Ben O’Brien, Tim Brooks, Natalie Bullock, David K. Butler, Gabriella Captur, Olivia Carr, Nicola Champion, Carmen Chan, Aneesh Chandran, Tom Coleman, Jorge Couto de Sousa, Xose Couto-Parada, Eleanor Cross, Teresa Cutino-Moguel, Silvia D’Arcangelo, Rhodri H. Davies, Brooke Douglas, Cecilia Di Genova, Keenan Dieobi-Anene, Mariana O. Diniz, Anaya Ellis, Karen Feehan, Malcolm Finlay, Marianna Fontana, Nasim Forooghi, Sasha Francis, Joseph M. Gibbons, David Gillespie, Derek Gilroy, Matt Hamblin, Gabrielle Harker, Georgia Hemingway, Jacqueline Hewson, Wendy Heywood, Lauren M. Hickling, Bethany Hicks, Aroon D. Hingorani, Lee Howes, Ivie Itua, Victor Jardim, Wing-Yiu Jason Lee, Melaniepetra Jensen, Jessica Jones, Meleri Jones, George Joy, Vikas Kapil, Caoimhe Kelly, Hibba Kurdi, Jonathan Lambourne, Kai-Min Lin, Siyi Liu, Aaron Lloyd, Sarah Louth, Mala K. Maini, Vineela Mandadapu, Charlotte Manisty, Áine McKnight, Katia Menacho, Celina Mfuko, Kevin Mills, Sebastian Millward, Oliver Mitchelmore, Christopher Moon, James Moon, Diana Muñoz Sandoval, Sam M. Murray, Mahdad Noursadeghi, Ashley Otter, Corinna Pade, Susana Palma, Ruth Parker, Kush Patel, Mihaela Pawarova, Steffen E. Petersen, Brian Piniera, Franziska P. Pieper, Lisa Rannigan, Alicja Rapala, Catherine J. Reynolds, Amy Richards, Matthew Robathan, Joshua Rosenheim, Cathy Rowe, Matthew Royds, Jane Sackville West, Genine Sambile, Nathalie M. Schmidt, Hannah Selman, Amanda Semper, Andreas Seraphim, Mihaela Simion, Angelique Smit, Michelle Sugimoto, Leo Swadling, Stephen Taylor, Nigel Temperton, Stephen Thomas, George D. Thornton, Thomas A. Treibel, Art Tucker, Ann Varghese, Jessry Veerapen, Mohit Vijayakumar, Tim Warner, Sophie Welch, Hannah White, Theresa Wodehouse, Lucinda Wynne, Dan Zahedi, Benjamin Chain, James C. Moon

**Affiliations:** ^1^ Department of Infectious Disease, Imperial College London, London, UK.; ^2^ Blizard Institute, Barts and the London School of Medicine and Dentistry, Queen Mary University of London, London, UK.; ^3^ UK Health Security Agency, Porton Down, UK.; ^4^ St Bartholomew’s Hospital, Barts Health NHS Trust, London, UK.; ^5^ Institute of Cardiovascular Science, University College London, London, UK.; ^6^ Department of Immunology and Inflammation, Imperial College London, London, UK.; ^7^ Lung Division, Royal Brompton and Harefield Hospitals, Guy’s and St Thomas’ NHS Foundation Trust, London, UK.

## Abstract

The Omicron, or Pango lineage B.1.1.529, variant of SARS-CoV-2 carries multiple spike mutations with high transmissibility and partial neutralizing antibody (nAb) escape. Vaccinated individuals show protection from severe disease, often attributed to primed cellular immunity. We investigated T and B cell immunity against B.1.1.529 in triple mRNA vaccinated healthcare workers (HCW) with different SARS-CoV-2 infection histories. B and T cell immunity against previous variants of concern was enhanced in triple vaccinated individuals, but magnitude of T and B cell responses against B.1.1.529 spike protein was reduced. Immune imprinting by infection with the earlier B.1.1.7 (Alpha) variant resulted in less durable binding antibody against B.1.1.529. Previously infection-naïve HCW who became infected during the B.1.1.529 wave showed enhanced immunity against earlier variants, but reduced nAb potency and T cell responses against B.1.1.529 itself. Previous Wuhan Hu-1 infection abrogated T cell recognition and any enhanced cross-reactive neutralizing immunity on infection with B.1.1.529.

At the end of 2021 the SARS-CoV-2 Omicron variant of concern (VOC) spread rapidly, displacing the prior most prevalent VOC, B.1.617.2 (Delta) (*1*, *2*). B.1.1.529 (Omicron) diverges more from the ancestral Wuhan Hu-1 sequence than other VOCs so far, with 36 coding mutations in spike, associated with high transmission, tendency to infect cells of the upper bronchus and presentation with flu-like symptoms (*3*–*5*). Across several studies, 2 or 3-dose vaccination is protective against severe disease and hospitalization, albeit with poor protection against transmission (*6*–*8*). A rationale for this high rate of breakthrough infections comes from mapping of virus neutralization using either post-vaccination immune sera or monoclonal antibodies, showing this to be the most antibody immune-evasive VOC, with titers generally reduced by 20-40-fold (*9*–*12*). The relative attenuation of severe symptoms in vaccinated compared to unvaccinated groups is likely attributable to the partial protection conferred by the residual neutralizing Ab (nAb) repertoire and the activation of primed B cell and T cell memory (*13*–*18*). In the present study we applied our ongoing analysis of a cohort of London healthcare workers (HCW) (*19*–*24*) to address two, key issues of B.1.1.529 (Omicron) immunity. First, following the earlier demonstration that people at this stage in the pandemic carry heterogeneous, immune-imprinted repertoires derived from their distinctive histories of infection and vaccination, we explored how these differences manifest in differential, cross-recognition of B.1.1.529 (Omicron) relative to other VOC, at the level of binding and neutralizing Ab, B cell and T cell immunity (*24*). Analyzing a London HCW cohort having detailed longitudinal, clinical, transcriptomic, and immunologic characterization, we considered the extent to which prior encounter with spike antigen through infection and vaccination shapes subsequent immunity to B.1.1.529 (Omicron) through immune imprinting. Second, when B.1.1.529 (Omicron) infections and reinfections have been so pervasive (*25*), it is possible that B.1.1.529 (Omicron) infection may confer a benign, live booster to vaccine immunity. Hence, we investigated the extent to which B.1.1.529 (Omicron) infection boosts cross-reactive B and T cell immunity against other VOC and itself.

## Results

### B cell immunity after three vaccine doses

**Fig. 1.**
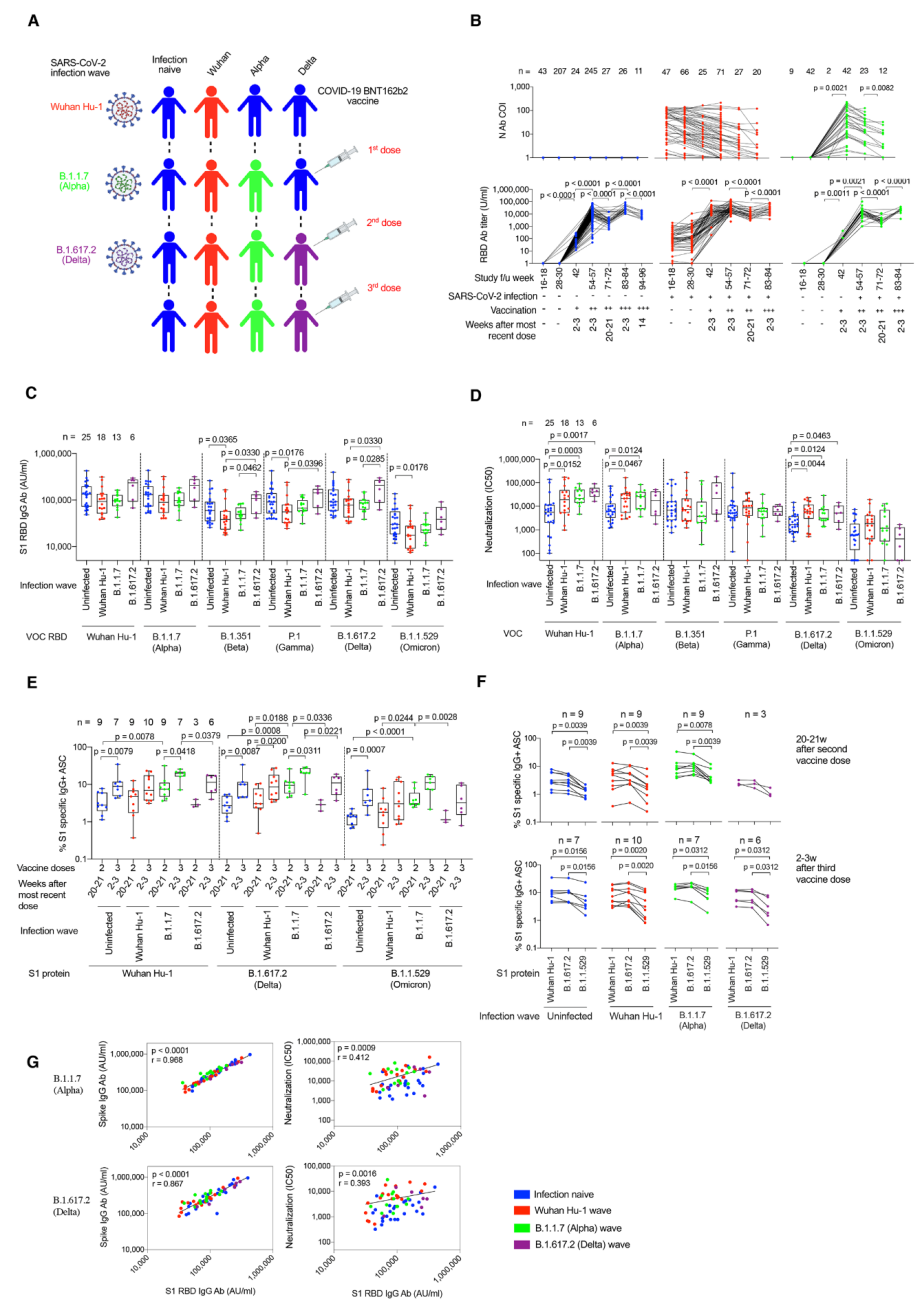
SARS-CoV-2 infection history alters Ab and B cell immunity in triple-vaccinated HCW. (**A**) Graphical summary depicting the SARS-CoV-2 infection and vaccination history of HCW studied. (**B**) Serum Ab binding against SARS-CoV-2 N (top panel) and ancestral Wuhan Hu-1 S1 RBD (bottom panel) in infection-naïve HCW (blue, n = 11-245) and HCW with laboratory confirmed SARS-CoV-2 infection during the Wuhan Hu-1 (red, n = 20-71) or B.1.1.7 (Alpha, green, n = 12-42) waves. Data are shown pre-vaccination and at defined timepoints following first, second and third dose of BNT162b2. (**C**) Serum S1 RBD Ab binding and (**D**) nAb IC50 against ancestral Wuhan Hu-1, B.1.1.7 (Alpha), B.1.351 (Beta), P.1 (Gamma), B.1.617.2 (Delta) and B.1.1.529 (Omicron) live virus 2-3w after the third vaccine dose in infection-naïve HCW (blue, n = 25) or HCW with laboratory confirmed SARS-CoV-2 infection during the ancestral Wuhan Hu-1 (red, n = 18), B.1.1.7 (Alpha, green, n = 13) or B.1.617.2 (Delta, purple, n = 6) waves. (**E**) Frequency of MBC specific for ancestral Wuhan Hu-1, B.1.617.2 (Delta) and B.1.1.529 (Omicron) spike S1 protein 20-21w after the second and 2-3w after the third vaccine dose in infection-naïve (blue, n = 7-9) or HCW infected by SARS-CoV-2 during the Wuhan Hu-1 (red, n = 9-10), B.1.1.7 (Alpha, green, n = 7-9) and B.1.617.2 (Delta, purple, n = 3-6) waves. (**F**) MBC frequency data plotted pairwise for individual HCW at 20-21w after second dose (top panel) or 2-3w after third dose (bottom panel). (**G**) Correlations between S1 RBD VOC and whole spike VOC Ab binding (left-hand panel) and nAb IC50 (right-hand panel) against B.1.1.7 (Alpha) and B.1.617.2 (Delta) in infection-naïve (blue, n = 25) HCW and HCW infected during the ancestral Wuhan Hu-1 (red, n = 18), B.1.1.7 (Alpha, green, n = 13) and B.1.617.2 (Delta, purple, n = 6) waves. Statistical tests were performed using Prism 9.0. [(B) to (E)] Mann-Whitney U test, (F) Wilcoxon matched-pairs signed rank test, (G) Spearman’s rank correlation. Ab, antibody; ASC, antibody secreting cells; AU, arbitrary units; COI, cut-off index; f/u; follow-up; HCW, health care workers; N, nucleocapsid; RBD, receptor binding domain; S1, subunit 1; MBC, memory B cell; VOC, variant of concern; w, weeks.

A London cohort of HCW were followed longitudinally from March 2020 to January 2022. HCW were identified with mild and asymptomatic SARS-CoV-2 infection by ancestral Wuhan Hu-1, B.1.1.7 (Alpha VOC), B.1.617.2 (Delta VOC) and then B.1.1.529 (Omicron VOC) during successive waves of infection and after first, second and third mRNA (BioNTech BNT162b2) vaccine doses (Fig. 1A, fig. S1, and table S1). We identified individuals with different combinations of SARS-CoV-2 infection and vaccination histories to study the impact of immune imprinting. N and S1 spike receptor binding domain (RBD) serology were monitored longitudinally (Fig. 1B). As previously reported, the third spike exposure boosted the majority of HCW above an S1 RBD titer of 1/10,000 binding antibody U/ml at 2-3 weeks after the most recent vaccine dose. By three vaccine doses antibody responses had plateaued, regardless of infection history (*24*).

In triple-vaccinated HCW 2-3 weeks after their third dose (table S1), we compared antibody titers against RBD (Fig. 1C), whole spike (fig. S2) and live virus nAb IC50 (Fig. 1D) for ancestral Wuhan Hu-1 and each of the VOC (table S2). We stratified the vaccinated HCW according to whether they were infection-naïve or had previously experienced infection with Wuhan Hu-1, B.1.1.7 (Alpha) or B.1.617.2 (Delta) (Fig. 1A). We found differences in immune imprinting indicating that those who were infected during the ancestral Wuhan Hu-1 wave showed a significantly reduced anti-RBD titer against B.1.351 (Beta), P.1 (Gamma) and B.1.1.529 (Omicron) compared to infection-naïve HCW (Fig. 1C). The hybrid immune groups that had experienced previous Wuhan Hu-1 and B.1.1.7 (Alpha) infection showed more potent nAb responses against Wuhan Hu-1, B.1.1.7 (Alpha) and B.1.617.2 (Delta) (Fig. 1D). However, cross-reactive S1 RBD IgG antibody and nAb IC50 against B.1.1.529 (Omicron) were significantly reduced compared to the other VOC irrespective of previous SARS-CoV-2 infection history (table S3 and Fig. 1, C and D).

Memory B cell (MBC) frequency against ancestral Wuhan Hu-1, B.1.617.2 (Delta) and B.1.1.529 (Omicron) S1 protein was boosted 2-3 weeks after the third vaccine dose compared to 20-21 weeks after the second vaccine dose (Fig. 1E). Irrespective of infection history, the MBC frequency against Wuhan Hu-1 and B.1.617.2 (Delta) S1 were similar, but significantly reduced against B.1.1.529 (Omicron) S1 2-3 weeks after the third vaccine dose [reduction in B.1.1.529 (Omicron) S1 MBC frequency compared to Wuhan Hu-1 S1 was 2-fold (p = 0.0156) for infection-naïve, 2.4-fold for Wuhan Hu-1 infection (p = 0.0020), 1.9-fold for B.1.1.7 infection (p = 0.0312) and 2.9-fold for B.1.617.2 infection (p = 0.0312)] and at 20-21 weeks after the second dose [reduction in B.1.1.529 (Omicron) MBC frequency compared to Wuhan Hu-1 was 2.5-fold (p = 0.0039) for infection naïve HCW and 2.2-fold (p = 0.0039), 2.0-fold (p = 0.0078) and 2.9-fold (p = 0.1250) for Wuhan Hu-1, B.1.1.7 and B.1.617.2 infection groups respectively] (Fig. 1F and table S4)

S1 RBD or whole spike antibody binding and live virus nAb IC50 correlated for B.1.1.7 (Alpha) and B.1.617.2 (Delta), but not for B.1.351 (Beta), P.1 (Gamma), B.1.1.529 (Omicron) indicating that antibody binding serology was a poor marker of nAb IC50 (Fig. 1G and fig. S3). Differences between VOC RBD and whole spike binding and nAb IC50 with live virus indicated that antibody targeting regions outside RBD/spike or conformational epitopes exposed only during infection may contribute to neutralization (*26*, *27*) (Fig. 1, C and D).

### T cell immunity after three vaccine doses

**Fig. 2.**
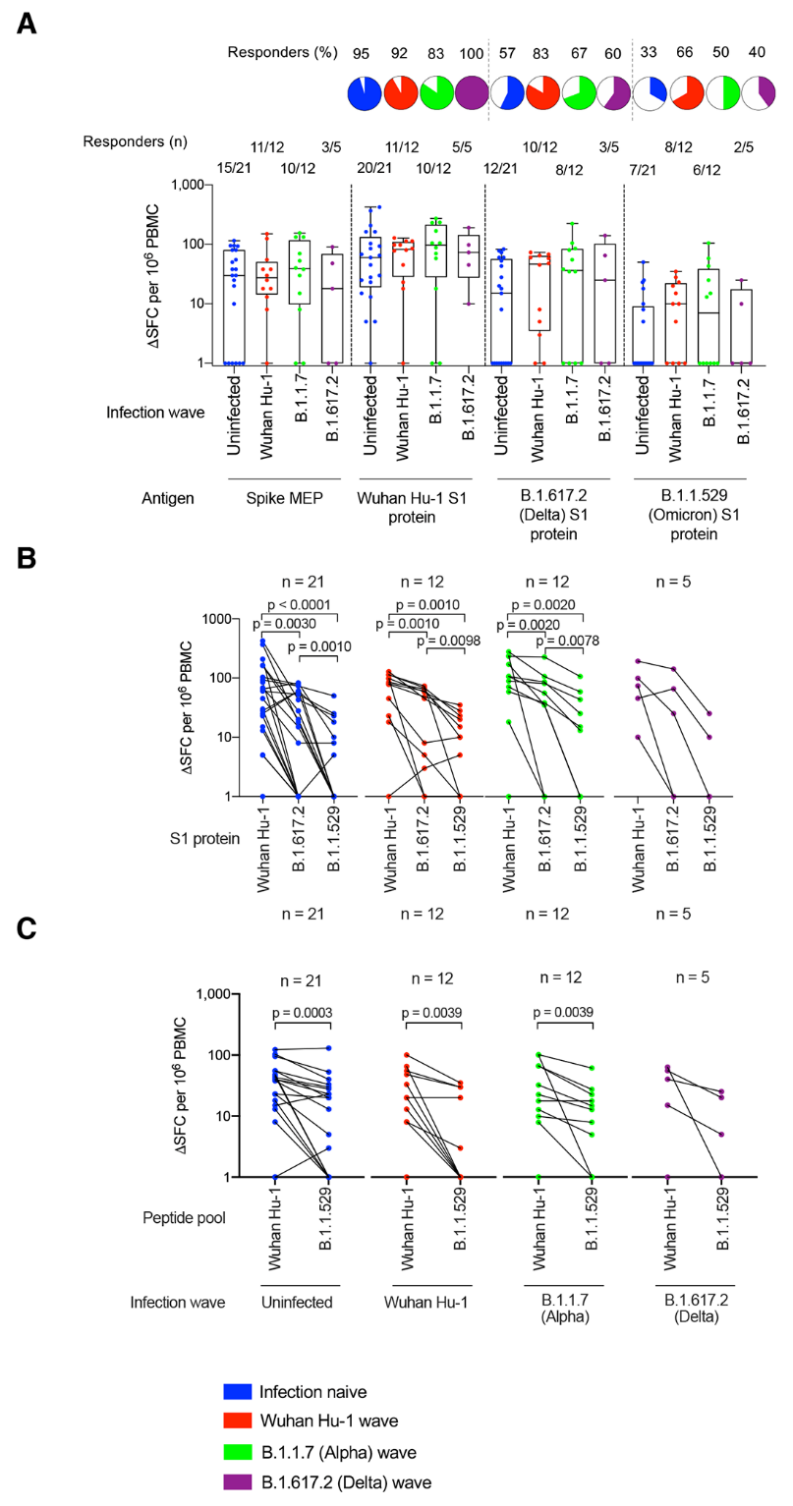
T cell cross-recognition of B.1.1.529 (Omicron) in triple-vaccinated HCW. (**A**) T cell responses against ancestral Wuhan Hu-1 spike MEP pool or ancestral Wuhan Hu-1, B.1.617.2 (Delta) and B.1.1.529 (Omicron) VOC S1 proteins in PBMC from infection-naïve HCW (blue) or HCW with laboratory confirmed SARS-CoV-2 infection during the ancestral Wuhan Hu-1 (red), B.1.1.7 (Alpha, green) and B.1.617.2 (Delta, purple) waves. PBMC were taken 2-3w after the third vaccine dose and T cell responses assessed by IFNγ ELISpot. Pie charts show the percent of responders with a detectable T cell response against each antigen. (**B**) Spike MEP pool and S1 protein T cell responses plotted pair-wise for each individual HCW. (**C**) T cell responses against peptide pools containing either the B.1.1.529 (Omicron) mutations found in SARS-CoV-2 spike or the equivalent original ancestral Wuhan Hu-1 sequences. PBMC from infection-naïve HCW (blue) or HCW infected during the ancestral Wuhan Hu-1 (red), B.1.1.7 (Alpha, green) and B.1.617.2 (Delta, purple) waves were stimulated by peptide pools containing the original Wuhan Hu-1 or B.1.1.529 sequences and plotted pair-wise. Statistical tests were performed using Prism 9.0. (A) Mann-Whitney U test, [(B) and (C)] Wilcoxon matched-pairs signed rank test. HCW, health care workers; MEP, mapped epitope peptide; PBMC, peripheral blood mononuclear cells; S1, subunit 1; SFC; spot forming cells; VOC, variant of concern; w, weeks.

We next compared T cell responses in triple-vaccinated HCW 2-3 weeks after the third dose, who were either infection-naïve or had been infected during the Wuhan Hu-1, B.1.1.7 (Alpha) or B.1.617.2 (Delta) waves (fig. S1 and table S1). We compared T cell immunity against a mapped epitope pool (MEP) of ancestral Wuhan Hu-1 spike peptides (table S5A) with spike S1 protein from ancestral Wuhan Hu-1 or S1 proteins containing the B.1.617.2 (Delta) or B.1.1.529 (Omicron) mutations. Measuring T cell responses against naturally processed epitopes from whole S1 protein antigen of ancestral Wuhan Hu-1, B.1.617.2 (Delta) or B.1.1.529 (Omicron) sequence allowed us to focus on immunodominant responses representative of those presented in real-life infection. T cell responses against Wuhan Hu-1 S1 protein mirrored those elicited by MEP stimulation, with the majority making a strong response (Fig. 2A). However, for S1 B.1.1.529 (Omicron) protein we found a significantly reduced magnitude of response. Overall, more than half (27/50; 54%) made no T cell response against S1 B.1.1.529 (Omicron) protein, irrespective of previous SARS-CoV-2 infection history, compared to 8% (4/50) that made no T cell response against ancestral Wuhan Hu-1 S1 protein (p < 0.0001, Chi-square test) (Fig. 2A). The fold-reduction in geometric mean of T cell response (SFC) against B.1.1.529 (Omicron) S1 compared to ancestral Wuhan Hu-1 S1 protein was 17.3-fold for infection-naïve HCW (blue, p < 0.0001), 7.7-fold for previously Wuhan Hu-1 infected (red, p = 0.001), 8.5-fold for previously B.1.1.7 (Alpha) infected (green, p = 0.002) and 19-fold for previously B.1.617.2 (Delta) infected (purple, p = 0.0625) HCW (Fig. 2B).

To investigate T cell recognition of VOC sequence mutations, we used a peptide pool specifically designed to cover all of the B.1.1.529 (Omicron) S1 and S2 spike mutations and a matched pool containing the Wuhan Hu-1 equivalent sequences (Fig. 2C and table S5B). T cell responses against the B.1.1.529 (Omicron) peptide pool were reduced compared to the Wuhan Hu-1 pool, irrespective of previous infection history [fold-reduction in T cell response against B.1.1.529 (Omicron) peptide pool compared to equivalent ancestral Wuhan Hu-1 peptide pool was 2.7-fold for infection-naïve (blue, p = 0.0003), 4.6-fold for previously Wuhan Hu-1 infected (red, p = 0.0039), 2.7-fold for previously B.1.1.7 (Alpha) infected (green, p = 0.0039) and 3.8-fold for previously B.1617.2 (Delta) infected (purple, p = 0.1250)] (Fig. 2C). In fact, 42% (21/50) of HCW make no T cell response at all against the B.1.1.529 (Omicron) VOC mutant pool (Fig. 2C).

Overall, our findings in triple-vaccinated HCW with different previous SARS-CoV-2 infection histories indicated that T cell cross-recognition of B.1.1.529 (Omicron) S1 antigen and peptide pool was significantly reduced.

T cell and nAb responses against B.1.1.529 (Omicron) were discordant and most (20/27, 74%) HCW with no T cell response against B.1.1.529 (Omicron) S1 made cross-reactive nAb against B.1.1.529 at an IC50 >195 (fig. S4).

### B.1.1.529 (Omicron) spike mutations encompass gain and loss of T cell epitopes

**Fig. 3.**
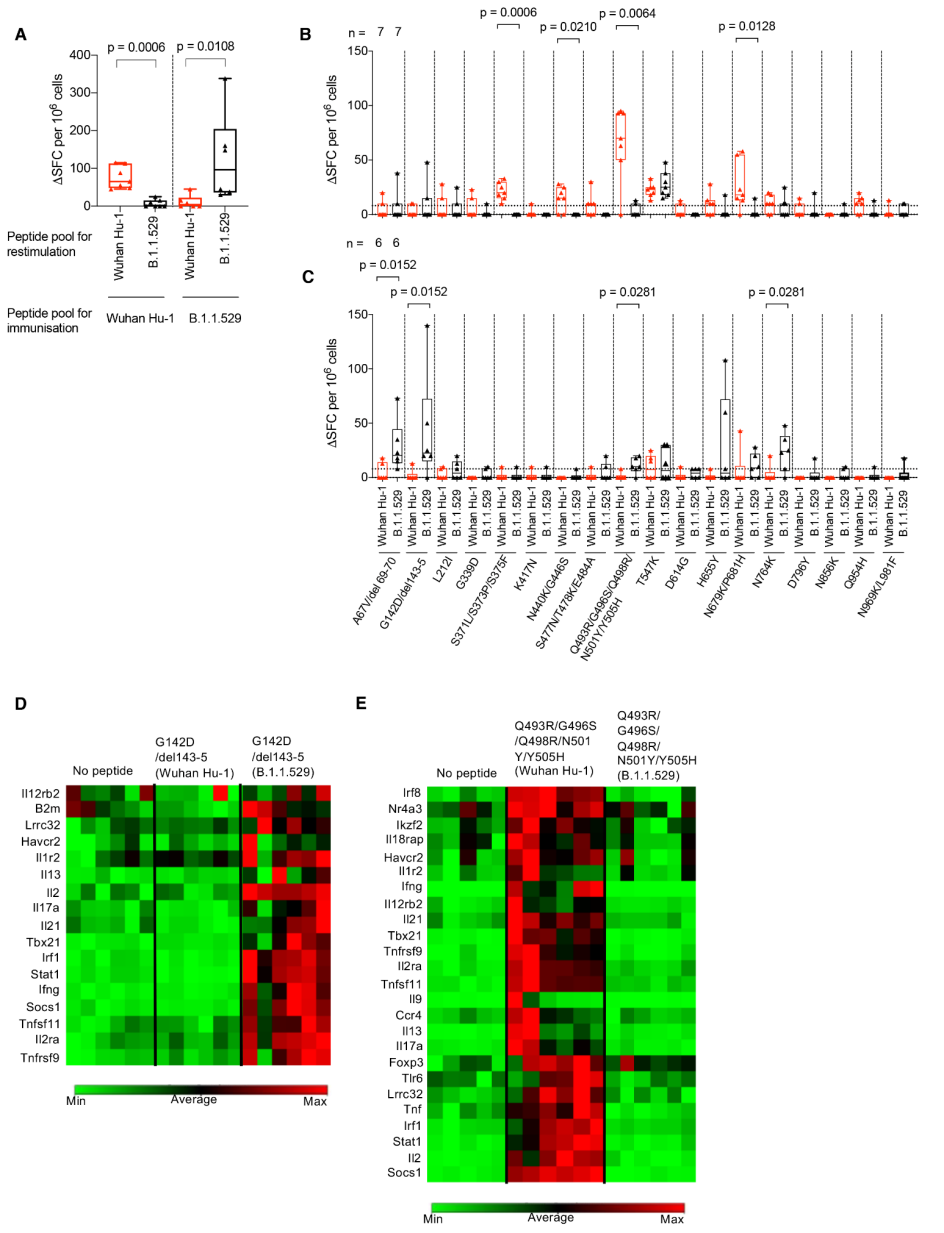
B.1.1.529 (Omicron) spike mutations alter T cell recognition. (**A**) HLAII transgenic mice carrying DRB1*0401 in the context of a homozygous knockout for murine H2-Aβ (7-8w) were immunized with either a B.1.1.529 (Omicron, n = 7) VOC pool of 18 peptides encompassing the Omicron sequence mutations or the ancestral Wuhan Hu-1 pool of peptides (n = 6) with the equivalent unmutated sequences. At d10 DLN cells were prepared from immunized mice and stimulated with either Wuhan Hu-1 (red) or B.1.1.529 (Omicron, black) peptide pools and T cell responses measured by IFNγ ELISpot. (**B**) IFNγ T cell responses were mapped against individual Wuhan Hu-1 (red) or B.1.1.529 (Omicron, black) peptides using DLN taken from Wuhan Hu-1 peptide pool (n = 7) or (**C**) B.1.1.529 (Omicron) peptide pool (n = 6) immunized mice. (**D** and **E**) Heatmaps showing relative gene expression of T cell activation markers in DLN cells taken from B.1.1.529 (Omicron) G142D/del143-5 peptide primed (D) (n = 6) or Wuhan Hu-1 Q493R/G496S/Q498R/N501Y/Y505H peptide primed (E) (n = 6) HLA-DRB1*04:01 transgenic mice. DLN cells were stimulated for 24h in vitro with 10 μg/ml Wuhan Hu-1 or B.1.1.529 (Omicron) variant peptide before RNA extraction. Genes shown in the heatmap are significantly up-regulated (p < 0.05) in Wuhan Hu-1 or B.1.1.529 (Omicron) variant peptide stimulated cells compared to no peptide control. Statistical tests were performed using Prism 9.0 or the Qiagen GeneGlobe data analysis tool for gene expression data. [(A) to (C)] Wilcoxon matched-pairs signed rank test, [(D) and (E)] Student’s *t* test. DLN, draining lymph node; SFC, spot forming cells; h, hours; VOC, variant of concern.

Due to the complexities inherent in mapping the effects of mutations in individual T cell epitopes across cohorts carrying heterogeneous HLA alleles, we mapped the differential recognition of the B.1.1.529 (Omicron) spike mutations using HLA-DRB1*04:01 transgenic mice (*23*, *24*) (Fig. 3). The peptide pool containing B.1.1.529 (Omicron) specific S1 and S2 spike mutations and its ancestral Wuhan Hu-1 equivalent pool showed differential, sequence-specific T cell priming by either ancestral Wuhan Hu-1 or B.1.1.529 (Omicron) sequence specific peptide pools (Fig. 3A and table S5B). That is, immunizing HLAII transgenic mice with either ancestral Wuhan Hu-1 or B.1.1.529 (Omicron) sequence specific peptide pools allowed us to investigate differential, sequence-specific T cell priming that occurs as a consequence of B.1.1.529 (Omicron) spike mutations. We showed that priming with one pool resulted in impaired responses to the other (Fig. 3A). We then looked at responses to individual HLA-DRB1*04:01 epitopes. Interestingly, while the B.1.1.529 (Omicron) mutations were associated in four instances with loss of a clear HLA-DR4-restricted T cell epitope (Fig. 3B: S371L/S373P/S375F, p = 0.0006; N440K/G446S, p = 0.0210; Q493R/G496S/Q498R/N501Y/Y505H, p = 0.0064; N679K/P681H, p = 0.0128), the mutated sequence epitopes in four instances led to de novo gain of Omicron-specific, HLA-DR4 T cell epitopes (Fig. 3C: A67V/del 69-70, p = 0.0152; G142D/del 143-5, p = 0.0152; Q493R/ G496S/Q498R/N501Y/Y505H; N679K/P681H, p = 0.0281; N764K, p = 0.0281). The G142D/del 143-5 mutation created a gain of function epitope, switching from a region not recognized by T cells, to one that induced a Th1/Th17 effector program (Fig. 3D). We have previously shown that the N501Y mutation converts a T cell effector-stimulating epitope to an inducer of immune regulation (*24*). This finding was confirmed and reiterated by the more extensive alterations in the Q493R/G496S/Q498R/N501Y/Y505H mutant epitope (Fig. 3E).

### B cell immunity after B.1.1.529 (Omicron) infection

**Fig. 4.**
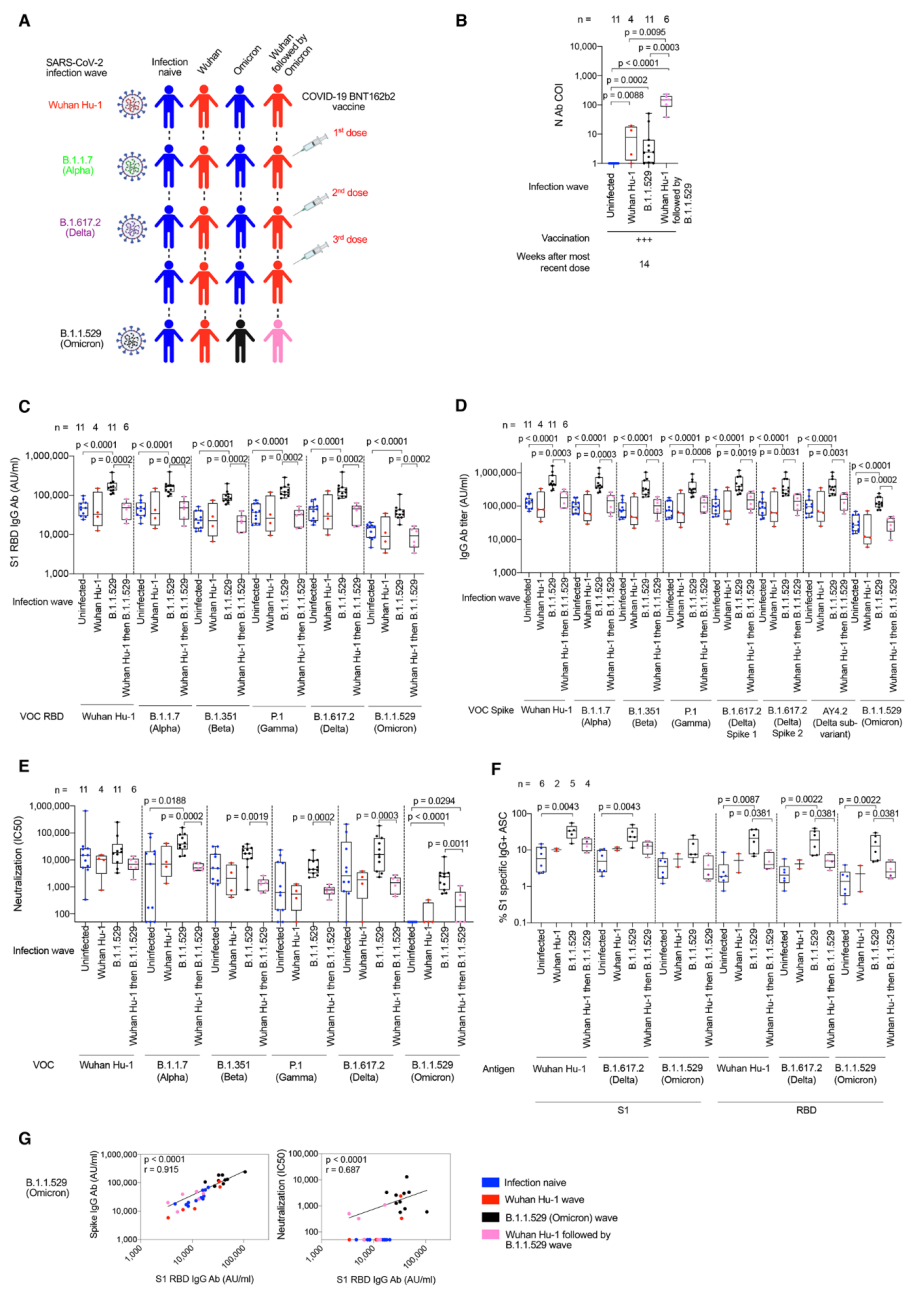
Ab and B cell immunity in triple-vaccinated HCW following infection during the B.1.1.529 (Omicron) wave. (**A**) Graphical summary depicting the SARS-CoV-2 infection and vaccination history of HCW studied during the B.1.1.529 (Omicron wave). (**B**) Serum Ab binding against SARS-CoV-2 N at 14w (median 14w, IQR 3w) after third vaccine dose in infection-naïve HCW (blue, n = 11) or in HCW with laboratory confirmed SARS-CoV-2 infection during the ancestral Wuhan Hu-1 (red, n = 4), B.1.1.529 (Omicron, black, n = 11) or Wuhan Hu-1 followed by B.1.1.529 (Omicron, pink, n = 6) infection waves. (**C**) Serum S1 RBD (VOC) Ab binding against ancestral Wuhan Hu-1, B.1.1.7 (Alpha), B.1.351 (Beta), P.1 (Gamma), B.1.617.2 (Delta) and B.1.1.529 (Omicron) proteins in infection-naïve HCW (blue, n = 11) or HCW previously infected during the ancestral Wuhan Hu-1 (red, n = 4), B.1.1.529 (Omicron, black, n = 11) or Wuhan Hu-1 followed by B.1.1.529 (Omicron, pink, n = 6) waves. (**D**) Serum Ab binding against ancestral Wuhan Hu-1, B.1.1.7 (Alpha), B.1.351 (Beta), P.1 (Gamma), B.1.617.2 (Delta), AY4.2 (Delta sub-variant) and B.1.1.529 (Omicron) whole spike proteins in infection-naïve HCW (blue, n = 11) or HCW previously infected during the ancestral Wuhan Hu-1 (red, n = 4), B.1.1.529 (Omicron, black, n = 11) or Wuhan Hu-1 followed by B.1.1.529 (Omicron, pink, n = 6) waves. (**E**) Neutralizing antibody IC50 against Wuhan Hu-1 or VOC live virus isolates in infection-naïve HCW (blue, n = 11) or HCW previously infected during the ancestral Wuhan Hu-1 (red, n = 4), B.1.1.529 (Omicron, black, n = 11) or Wuhan Hu-1 followed by B.1.1.529 (Omicron, pink, n = 6) waves. (**F**) Frequency of MBC specific for ancestral Wuhan Hu-1, B.1.617.2 (Delta) and B.1.1.529 (Omicron) S1 and RBD binding proteins in infection-naïve HCW (blue, n = 11) or HCW previously infected during the ancestral Wuhan Hu-1 (red, n = 4), B.1.1.529 (Omicron, black, n = 11) or Wuhan Hu-1 followed by B.1.1.529 (Omicron, pink, n = 6) waves. (**G**) Correlation between whole spike and S1 RBD Ab binding (left-hand panel) or nAb IC50 and S1 RBD Ab binding (right-hand panel) for B.1.1.529 (Omicron) VOC in infection-naïve (blue, n = 11) or HCW infected during the ancestral Wuhan Hu-1 (red, n = 4), B.1.1.529 (Omicron, black, n = 11) or Wuhan Hu-1 followed by B.1.1.529 (Omicron, pink, n = 6) waves. All data shown is from samples taken at 14w (median 14w, IQR 3w) after third vaccine dose. Statistical tests were performed using Prism 9.0. [(B) to (F)] Mann-Whitney U test, (G) Spearman’s rank correlation. Ab, antibody; ASC, antibody secreting cells; AU, arbitrary units; COI, cut-off index; HCW, health care workers; IQR, inter-quartile range; N, nucleocapsid; RBD, receptor binding domain; S1, subunit 1; MBC, memory B cell; VOC, variant of concern; w, weeks.

Next, we studied triple-vaccinated HCW 14-weeks after their third dose, who had suffered breakthrough infection during the B.1.1.529 (Omicron) wave. These individuals were compared to infection-naïve and prior Wuhan Hu-1 infected HCW that had escaped B.1.1.529 (Omicron) wave infection (Fig. 4A, tables S6 and S7, and fig. S1). Wuhan Hu-1 prior infected HCW that were also infected during the B.1.1.529 (Omicron) wave showed the highest N antibody binding (Fig. 4B and tables S6 and S7). Previously infection-naïve triple-vaccinated HCW made significantly increased cross-reactive antibody binding responses against all VOC and B.1.1.529 (Omicron) itself after infection during the B.1.1.5129 (Omicron) wave: S1 RBD (Fig. 4C and table S2), whole spike (Fig. 4D and table S2) and nAb IC50 (Fig. 4E). However, antibody binding and nAb IC50 were attenuated against B.1.1.529 (Omicron) itself with a 4.5-fold reduction in S1 RBD binding (p = 0.001) and 10.1-fold reduction in nAb IC50 (p = 0.002) against B.1.1.529 compared to ancestral Wuhan Hu-1. Thus, infection during the B.1.1.529 (Omicron) wave produced potent cross-reactive antibody immunity against all VOC, but less so against B.1.1.529 (Omicron) itself.

Importantly, triple-vaccinated, infection-naïve HCW that were not infected during the B.1.1.529 (Omicron) wave made no nAb IC50 response against B.1.1.529 (Omicron) 14 weeks after the third vaccine dose indicating rapid waning of the nAb IC50 from a mean value of 1400 at 2-3w falling to 0 at 14w after the third dose (p = 0.0312) (Fig. 4E and fig. S5A).

HCW with a history of prior Wuhan Hu-1 infection that were also infected during the B.1.1.529 (Omicron) wave showed no increase in cross-reactive S1 RBD (Fig. 4C) or whole spike (Fig. 4D) antibody binding or live virus nAb IC50 (Fig. 4E and fig. S5B) against B.1.1.529 (Omicron) or any other VOC, despite having made a higher N antibody response (Fig. 4B). Thus, B.1.1.529 (Omicron) infection can boost binding and nAb responses against itself and other VOC in triple-vaccinated previously uninfected infection naïve HCW, but not in the context of immune imprinting following prior Wuhan Hu-1 infection. Immune imprinting by prior Wuhan Hu-1 infection completely abrogated any enhanced nAb responses against B.1.1.529 (Omicron) and other VOC (Fig. 4E).

Increased MBC frequency against ancestral Wuhan Hu-1, B.1.617.2 (Delta) and B.1.1.529 (Omicron) S1 and RBD proteins were observed in previously infection naïve HCW infected during the B.1.1.529 (Omicron) wave (Fig. 4F). This was not the case for HCW that had been previously infected during the first Wuhan Hu-1 wave and then again during the B.1.1.529 (Omicron) wave (Fig. 4F).

In summary, B.1.1.529 (Omicron) infection resulted in enhanced, cross-reactive Ab responses against all VOC tested in the three-dose vaccinated infection-naïve HCW, but not those with previous Wuhan Hu-1 infection, and less so against B.1.1.529 (Omicron) itself (Fig. 4, C to E). In line with this, MBC frequency against Wuhan Hu-1, B.1.617.2 (Delta) and B.1.1.529 (Omicron) S1 and RBD proteins increased in three-dose vaccinated, infection-naïve individuals, but not those imprinted by previous Wuhan Hu-1 infection (Fig. 4F).

S1 RBD or whole spike antibody binding and live virus nAb IC50 correlated for B.1.1.529 (Omicron) (r = 0.687, p < 0.0001) and all the VOC tested (r > 0.539), but there was significant discordance in that many of the HCW recording no detectable live virus nAb IC50 against B.1.1.529 (Omicron) recorded S1 RBD (Omicron) binding serology ranging from 3412 to 20,484 indicating that S1 RBD (VOC) antibody binding serology could be misleading and a poor marker of nAb (Fig. 4G and fig. S6).

### T cell immunity after B.1.1.529 (Omicron) infection

**Fig. 5.**
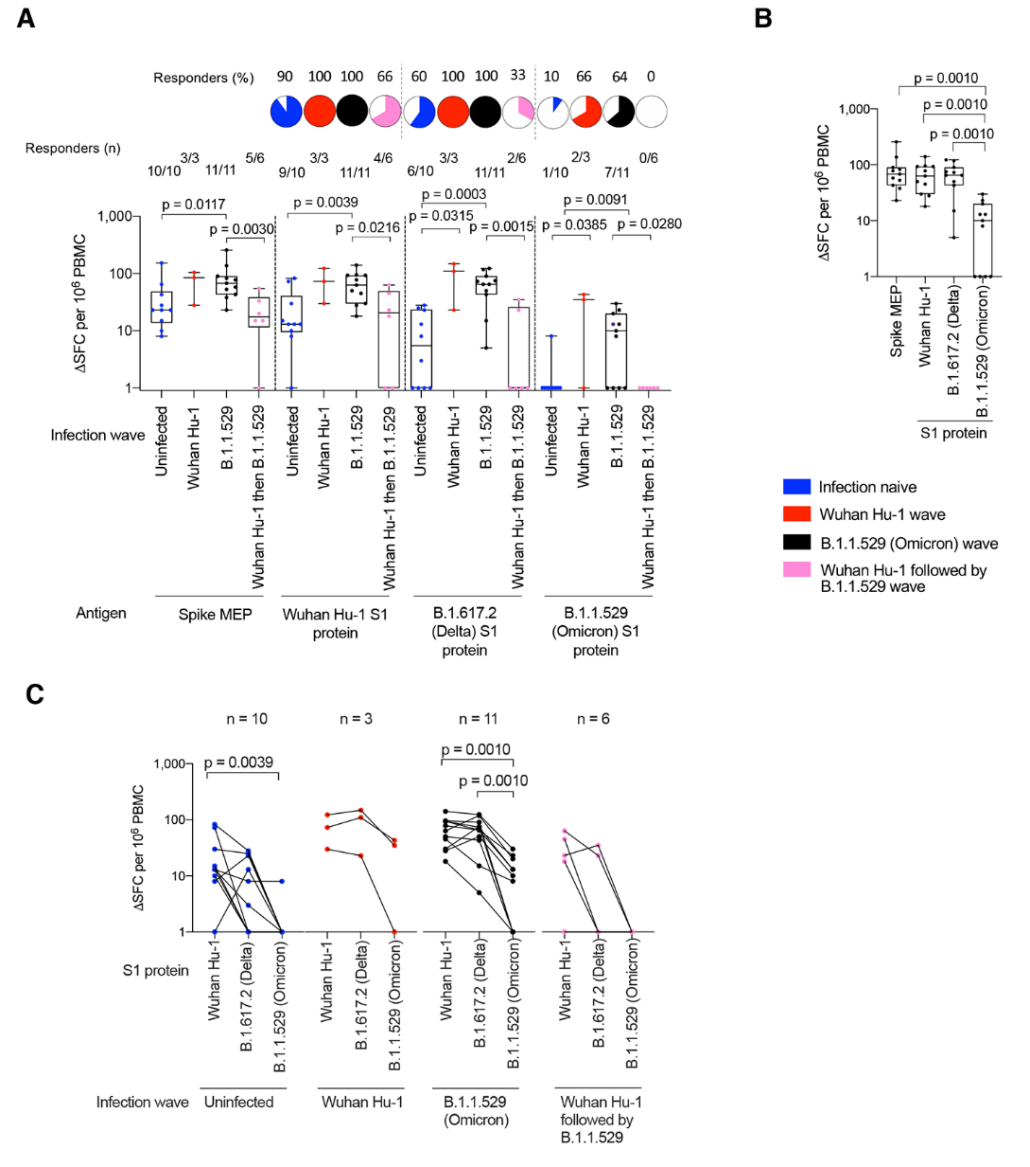
T cell responses in triple-vaccinated HCW infected during the B.1.1.529 (Omicron) wave. (**A**) T cell responses against ancestral Wuhan Hu-1 spike MEP pool or Wuhan Hu-1, B.1.617.2 (Delta) and B.1.1.529 (Omicron) VOC S1 protein for PBMC taken from infection-naïve HCW (blue, n = 10) or HCW with laboratory confirmed SARS-CoV-2 infection during the Wuhan Hu-1 (red, n = 3), B.1.1.529 (Omicron, black, n = 11) or Wuhan Hu-1 followed by B.1.1.529 (Omicron, pink, n = 6) waves. PBMC were taken 14w (median 14w, IQR 3w) after the third vaccine dose and T cell responses assessed by IFNγ ELISpot. Pie charts show the percent that had a detectable T cell response against each antigen. (**B**) T cell responses against spike MEP pool and S1 VOC protein for previously infection naïve triple-vaccinated HCW infected during the B.1.1.529 (Omicron, black, n = 11) wave. (**C**) T cell responses against ancestral Wuhan Hu-1, B.1.617.2 (Delta) and B.1.1.529 (Omicron) S1 proteins plotted pair-wise for infection-naïve HCW (blue, n = 10) or HCW with laboratory confirmed SARS-CoV-2 infection during the Wuhan Hu-1 (red, n = 3), B.1.1.529 (Omicron, black, n = 11) or Wuhan Hu-1 followed by B.1.1.529 (Omicron, pink, n = 6) waves. Statistical tests were performed using Prism 9.0. (A) Mann-Whitney U test, [(B) and (C)] Wilcoxon matched-pairs signed rank test. HCW, health care workers; IQR, inter-quartile range; MEP, mapped epitope peptide; PBMC, peripheral blood mononuclear cells; S1, subunit 1; SFC; spot forming cells; VOC, variant of concern; w, weeks.

We next explored T cell immunity following breakthrough infection during the B.1.1.529 (Omicron) wave. Fourteen weeks after the third dose (9/10, 90%) of triple-vaccinated, previously infection-naïve HCW showed no cross-reactive T cell immunity against B.1.1.529 (Omicron) S1 protein (Fig. 5A).

The T cell response against B.1.1.529 (Omicron) S1 protein following infection during the B.1.1.529 (Omicron) wave of previously infection-naïve HCW was significantly reduced compared to Wuhan Hu-1 S1 and B.1.617.2 (Delta) S1 in triple-vaccinated HCW [geometric mean, 57, 50 and 6 SFC for Wuhan Hu-1, B.1.617.2 (Delta) and B.1.1.529 (Omicron) S1 proteins respectively, p = 0.001)] (Fig. 5B). HCW infected during the B.1.1.529 (Omicron) wave showed similar T cell responses against spike MEP, ancestral Wuhan Hu-1 S1 and B.1.617.2 (Delta) S1 proteins, but significantly reduced T cell responses against B.1.1.529 (Omicron) S1 protein [reduction in geometric mean of T cell response (SFC) against VOC S1 protein compared to that for Wuhan Hu-1 S1: 1.1-fold reduction for B.1.617.2 S1, p = 0.6836; 10-fold reduction for B.1.1.529 S1, p = 0.001] (Fig. 5B). Thus, although breakthrough infection in triple-vaccinees during the Omicron infection wave boosted cross-reactive T cell immune recognition against the spike MEP pool (p = 0.0117), ancestral Wuhan Hu-1 (p = 0.0039), B.1.617.2 (Delta) (p = 0.0003) and B.1.1.529 (Omicron) (Fig. 5A), the T cell response against B.1.1.529 (Omicron) S1 protein itself compared to spike MEP (p = 0.001), Wuhan Hu-1 (p = 0.001), and B.1.617.2 (Delta) (p = 0.001) was significantly reduced (Fig. 5, B and C).

Importantly, none (0/6) of HCW with a previous history of SARS-CoV-2 infection during the Wuhan Hu-1 wave responded to B.1.1.529 (Omicron) S1 protein (Fig. 5A). This suggests that, in this context, B.1.1.529 (Omicron) infection was unable to boost T cell immunity against B.1.1.529 (Omicron) itself; immune imprinting from prior Wuhan Hu-1 infection resulted in absence of a T cell response against B.1.1.529 (Omicron) S1 protein. These findings were further highlighted in paired data showing the fall in T cell response in individual HCWs across the three antigens: on an individual basis, most HCW retained T cell recognition of B.1.617.2 S1, but commonly showed impaired T cell recognition of B.1.1.529 S1 (Fig. 5C). Taken together with the data shown in Fig. 4, the findings show consistently that people initially infected by Wuhan Hu-1 in the first wave and then reinfected during the B.1.1.529 (Omicron) wave do not boost T cell immunity against B.1.1.529 (Omicron) at the level of nAb and T cell recognition.

### Prior infection differentially imprints Omicron T and B cell immunity

**Fig. 6.**
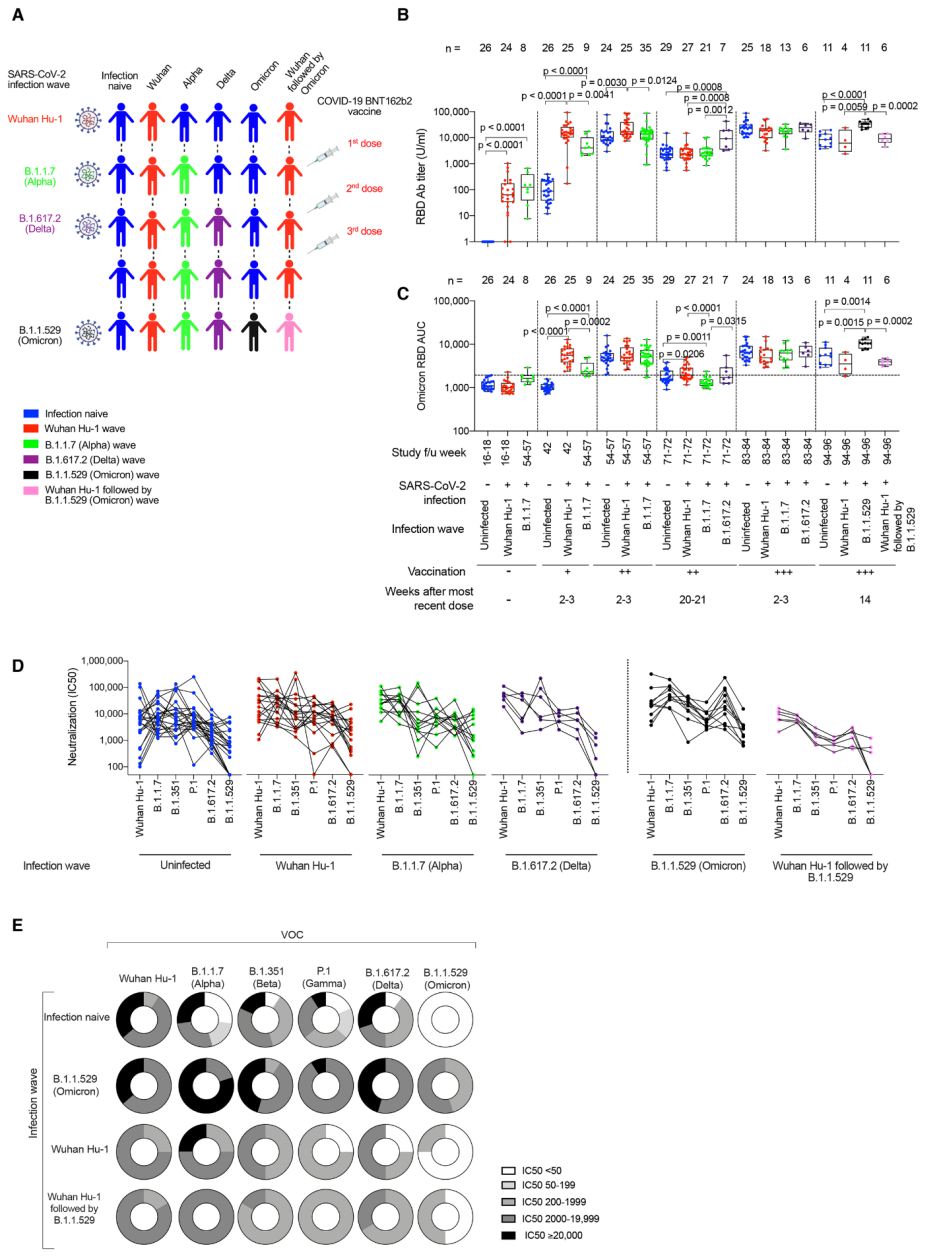
SARS-CoV-2 infection imprints differential Ab cross-reactivity to VOC. (**A**) Graphical summary depicting the SARS-CoV-2 infection and vaccination history of HCW studied. Infection naïve HCW are indicated in blue. HCW infected during the different waves are indicated as follows: ancestral Wuhan Hu-1 (red), B.1.1.7 (Alpha, green) and B.1.617.2 (Delta, purple), B.1.1.529 (Omicron, black) and Wuhan Hu-1 followed by B.1.1.529 (Omicron, pink). (**B** and **C**) Serum Ab binding against ancestral Wuhan Hu-1 S1 RBD (B) and B.1.1.529 (Omicron) S1 RBD (C) in infection-naïve HCW (blue, n = 11-29) or in HCW with laboratory confirmed SARS-CoV-2 infection during the ancestral Wuhan Hu-1 (red, n = 4-27), B.1.1.7 (Alpha, green, n = 8-35), B.1.617.2 (Delta, purple, n = 6-7), B.1.1.529 (Omicron, black, n = 11) or Wuhan Hu-1 followed by B.1.1.529 (Omicron, pink, n = 6) waves. Data are shown pre-vaccination and at timepoints following first, second and third dose of vaccine. (**D**) Cross-reactive nAb IC50 against ancestral Wuhan Hu-1, B.1.1.7 (Alpha), B.1.351 (Beta), P.1 (Gamma), B.1.617.2 (Delta) and B.1.1.529 (Omicron) live virus 2-3w after third vaccine dose in infection-naïve HCW (blue, n = 24) or HCW with laboratory confirmed SARS-CoV-2 infection during the Wuhan Hu-1 (red, n = 18), B.1.1.7 (Alpha, green, n = 13) or B.1.617.2 (Delta, purple, n = 6) waves and at 14w (median 14w, IQR 3w) after third vaccine dose in HCW with laboratory confirmed SARS-CoV-2 during the B.1.1.529 (Omicron, black, n = 11) or Wuhan Hu-1 followed by B.1.1.529 (Omicron, pink, n = 6) waves. Data are plotted pair-wise for individual HCW. (**E**) Doughnuts showing the relative proportion of HCW with nAb IC50 of <50 (white), 50-199 (25% gray), 200-1,999 (50% gray), 2,000-19,999 (75% gray) and ≥ 20,000 (black) against ancestral Wuhan Hu-1, B.1.1.7 (Alpha), B.1.351 (Beta), P.1 (Gamma), B.1.617.2 (Delta) and B.1.1.529 (Omicron) live virus in infection-naïve HCW (n = 11) or HCW with laboratory confirmed infection during the B.1.1.529 (Omicron, n = 11), Wuhan Hu-1 (n = 4) or Wuhan Hu-1 followed by B.1.1.529 (Omicron, n = 6) waves at 14w (median 14w, IQR 3w) after the third vaccine dose. Statistical tests were performed using Prism 9.0. [(B) and (C)] Mann-Whitney U test. Ab, antibody; nAb neutralizing antibody; HCW, health care workers; IQR, inter-quartile range; RBD, receptor binding domain; VOC, variant of concern; w, weeks.

To investigate in more detail the impact of prior SARS-CoV-2 infection on immune imprinting, we further explored responses in our longitudinal HCW cohort (Fig. 6A and fig. S1). We looked initially at the S1 RBD (ancestral Wuhan Hu-1 and Omicron VOC) antibody binding responses across the longitudinal cohort at key vaccination and SARS-CoV-2 infection timepoints, exploring how different exposure imprinted differential cross-reactive immunity and durability. This revealed that at 16-18 weeks after Wuhan Hu-1 infection or B.1.1.7 (Alpha) infection, unvaccinated HCW showed no detectable cross-reactive S1 RBD binding antibodies against B.1.1.529 (Omicron) (Fig. 6C).

Hybrid immunity (the combination of prior infection and a single vaccine dose) significantly increased the S1 RBD binding antibodies against B.1.1.529 (Omicron) (p < 0.0001) compared to responses of infection-naïve HCW, which were undetectable after a single vaccine dose. This increase was significantly greater for prior Wuhan Hu-1 than B.1.1.7 (Alpha) infected HCW (p < 0.0002) (Fig. 6, B and C).

Two to three weeks after two vaccine doses there was a levelling up of S1 RBD B.1.1.529 (Omicron) binding antibody, such that infection-naïve, prior Wuhan Hu-1 and B.1.1.7 (Alpha) infected HCW made similar responses (Fig. 6, B and C).

However, 20-21 weeks after the second vaccine dose, differential B.1.1.529 (Omicron) RBD Ab waning was noted with almost all (19/21) of the HCW infected during the second B.1.1.7 (Alpha) wave no longer showing detectable cross-reactive antibody against B.1.1.529 (Omicron) RBD (Fig. 6C). This was distinct from HCW infected by Wuhan Hu-1 during the first wave where there was a significantly higher cross-protective antibody response against B.1.1.529 (Omicron) RBD (p < 0.0001) (Fig. 6C). This indicates a profound differential impact of immune imprinting on B.1.1.529 (Omicron) specific immune antibody waning between HCW infected by Wuhan Hu-1 and B.1.1.7 (Alpha) as this differential is not seen in Ab responses to ancestral WuhanHu-1 spike S1 RBD (Fig. 6B).

Again, there was a levelling up back to similar B.1.1.529 (Omicron) RBD binding across infection-naïve and previously infected HCW [Wuhan Hu-1, B.1.1.7 (Alpha) and B.1.617.2 (Delta)] 2-3 weeks after the third vaccine dose (Fig. 6, B and C).

Fourteen weeks after the third vaccine dose previously infection-naïve HCW infected during the B.1.1.529 (Omicron) wave showed increased S1 RBD B.1.1.529 (Omicron) binding responses, but prior Wuhan Hu-1 infected HCW did not, indicating that prior Wuhan Hu-1 infected individuals were immune imprinted to not boost antibody binding responses against B.1.1.529 (Omicron) despite having been infected by B.1.1.529 (Omicron) itself (Fig. 6C).

In fact, infection during the B.1.1.529 (Omicron) wave imprinted a consistent relative hierarchy of cross-neutralization immunity against VOC across different individuals with potent cross-reactive nAb responses against B.1.1.7 (Alpha), B.1.351 (Beta) and B.1.617.2 (Delta) (Fig. 6, D and E). Comparative analysis of nAb potency for cross-neutralization of VOC emphasized the impact of immune imprinting which effectively abrogates the nAb responses in those vaccinated HCW infected during the first wave and then reinfected during the B.1.1.529 (Omicron) wave. The doughnuts highlight the extent to which the relative potency of nAb responses are attenuated in prior Wuhan Hu-1 infected HCW (Fig. 6E).

## Discussion

At this stage in the pandemic there is a view that the global spread of B.1.1.529 (Omicron), through its association with a relatively milder disease phenotype and, possibly, a potential to boost vaccine immunity, may herald the transition into a new, endemic relationship (*28*). The case for vaccine-mediated immune preconditioning as key mediator of the attenuated phenotype is complex: while functional neutralization by vaccine-primed sera is considerably blunted against B.1.1.529 (Omicron), three-dose vaccination efficacy against symptomatic disease holds up, in the 50-70% range (*6*–*8*). It has thus been proposed that immune protection may be supported by maintenance of relatively high T cell response frequencies to viral epitopes unperturbed by loss of antibody epitopes (*13*–*18*). A rationale for this T cell mediated protection comes from animal studies showing the direct ability of SARS-CoV-2 specific T cells to curtail lung viral loads (*29*). This raised two key questions with respect to understanding and management of this wave: (i) considering the very diverse patterns of antiviral immunity shown by ourselves and others to be determined by differential immune imprinting, how would differences in antigen exposure through infection and vaccination alter immune responses against B.1.1.529 (Omicron) at the level of binding antibody and nAb, MBC and T cell responses? (ii) Is the immune response following infection during the B.1.1.529 (Omicron) wave primed and fully available to support protective immunity? We examined immunity to B.1.1.529 (Omicron) in a longitudinal HCW cohort, considering cross-reactive immunity primed by the varied spike exposures of 3-dose vaccination with or without hybrid immunity from any of the Wuhan Hu-1, B.1.1.7 (Alpha) or B.1.617.2 (Delta) infection waves, and then, the additive effective of actual infection during the B.1.1.529 (Omicron) wave. In the first part of this paper we report patterns of response in differentially imprinted, triple-vaccinated HCW. In the second part of the paper we consider immune responses in those who went on to suffer infection during the B.1.1.529 (Omicron) infection wave despite triple-vaccination. There were several unexpected findings. While it is known that cross-reactive antibody recognition is compromised by the mutations in B.1.1.529 (Omicron), it was surprising that this was so profoundly exacerbated by differential imprinting in those having had prior infection with either Wuhan Hu-1 or B.1.1.7 (Alpha). This adds an important dimension to global control of B.1.1.529 (Omicron) in light of the impact B.1.1.7 (Alpha) has had on the global pandemic: by May 2021 B.1.1.7 (Alpha) accounted for 67% of all cases across 149 countries (*30*). That previous SARS-CoV-2 infection history can imprint such a profound, negative impact on subsequent protective immunity is an unexpected consequence of COVID-19. While the notion that, generally, hybrid priming by infection and vaccination enhances immunity is widely agreed (*22*), imprinted patterns such as the specific combination of vaccination with infection during the first ancestral Wuhan Hu-1 wave followed by the B.1.1.529 (Omicron) wave requires an additional term—“hybrid-immune-damping.” Molecular characterization of the precise mechanism underpinning repertoire shaping from a combination of Wuhan Hu-1 or B.1.1.7 (Alpha) infection and triple-vaccination using ancestral Wuhan Hu-1 sequence, impacting immune responses to subsequent VOCs, will require detailed analysis of differential immune repertoires and their structural consequences. The impact of differential imprinting was seen just as profoundly in T cell recognition of B.1.1.529 (Omicron) S1, which was not recognized by T cells from any triple-vaccinated HCW who were initially infected during the Wuhan Hu-1 wave and then re-infected during the B.1.1.529 (Omicron) wave. Importantly, while B1.1.529 (Omicron) infection in triple-vaccinated previously uninfected individuals could indeed boost antibody, T cell and MBC responses against other VOC, responses to itself were reduced. This relatively poor immunogenicity against itself may help to explain why frequent B.1.1.529 (Omicron) reinfections with short time intervals between infections are proving a novel feature in this wave. It also concurs with observations that mRNA vaccination carrying the B.1.1.529 (Omicron) spike sequence (Omicron third-dose after ancestral sequence prime/boosting) offers no protective advantage (*31*). Initial studies using acute serum samples following B.1.1.529 (Omicron) infection had indicated poor immunogenicity and a tendency to elicit only Omicron-specific responses in the unvaccinated and broader responses in those imprinted following COVID-19 vaccination (*32*, *33*), including unexpected patterns of combinations that appeared to ablate neutralizing responses to previously seen VOC (*33*).

Our T cell analysis, which depended on processing of immunodominant epitopes from whole antigen, revealed a more profound deficit than others. Studies in which T cell responses of vaccinees against spike peptide megapools are screened show that, while there may be a 20% drop in response due to lost epitopes across the entire sequence, most of the response is maintained (*13*–*15*, *17*), albeit with a significant minority showing a completely ablated CD8 response to Omicron peptide pools (*17*). Other studies show that around a fifth of responders to peptide panels have a 50-70% drop in T cell response (*16*). Our approach was to evaluate T cell recognition using the dual approach of mapped epitope pools spanning the mutated regions and also, whole, naturally processed antigen. We found the greatest impairment of T cell recognition when looking at epitope recognition after processing of whole antigen. Naturally processed epitopes from uptake of whole antigen would generally be considered more representative of the real-life situation and nearer to HLA-ligandome studies than synthetic megapools of several hundred overlapping peptides which have the potential to drown out physiological response patterns under the noise of responses from cryptic epitopes that may not feature in real-life natural responses. That is, megapool approaches can, by their nature, underestimate the extent of response ablation. The natural HLA-ligandome of peptides shown to be elicited by natural processing and HLAII presentation only partially overlaps epitopes mapped from overlapping synthetic peptide panels (*34*, *35*). Our immunization of mice with B.1.1.529 mutant epitopes confirmed that de novo T cell response repertoire can be elicited, but this is not necessarily the same as that generated during live infection.

In summary, these studies have shown that the high global prevalence of B.1.1.529 (Omicron) infections and reinfections likely reflects considerable subversion of immune recognition at both the B, T cell, antibody binding and nAb level, although with considerable differential modulation through immune imprinting. Some imprinted combinations, such as infection during the Wuhan Hu-1 and Omicron waves, confer particularly impaired responses.

## Materials and methods

### Study subjects

A total of 731 adult HCW were recruited into the COVIDsortium bioresource in March 2020 (*19*–*24*) (fig. S1). A cross-sectional case controlled sub-study of 136 HCW recruited 16-18 weeks after March 2020 UK lockdown reported immunity to SARS-CoV-2 infection during the UK 1st wave (Wuhan Hu-1) (*19*). SARS-CoV-2 infection was determined by baseline and weekly nasal RNA stabilizing swabs and Roche cobas® SARS-CoV-2 reverse transcriptase polymerase chain reaction (RT-PCR) test and baseline and weekly Antibody testing for S1 using the IgG EUROIMMUN enzyme-linked immunosorbent assay (ELISA) and nucleocapsid using the ROCHE Elecsys electrochemiluminescence immunoassay (ECLIA). Antibody ratios >1.1 were deemed positive for the EUROIMMUN SARS-CoV-2 ELISA and >1 was considered test positive for the ROCHE Elecsys anti-SARS-CoV-2 ECLIA, as evaluated by UK Health Security Agency (UKHSA), Porton Down, UK. A cross-sectional, case-controlled vaccine sub-study cohort of 51 HCW at a mean timepoint of 22d (±2d SD) after administration of the first dose of BNT162b2 vaccines reported immunity to vaccination in individuals with and without a history of prior SARS-CoV-2 infection during the Wuhan Hu-1 wave (*23*)*.* The vaccine sub-study recruited HCW previously enrolled in the 16-18 week sub-study. It included 25 HCW (mean age 44y, 60% male) with previous lab-defined SARS-CoV-2 infection and 26 HCW (mean age 41y, 54% male) with no laboratory evidence of SARS-CoV-2 infection throughout the initial 16-week longitudinal follow up. HCW were followed up longitudinally (n = 51) at a median timepoint of 20 days (7, IQR) after administration of the second dose of BNT162b2 (*24*). An additional 358 HCW recruited at 55-57 weeks follow-up, 53 of whom were infected by the B.1.1.7 (Alpha) VOC during the second UK wave (*24*). At 71-72 weeks follow-up, 80 two dose vaccinated HCW were re-recruited that were either SARS-CoV-2 infection naïve (n = 27) or had been infected by Wuhan Hu-1 during the first wave (n = 31) or B.1.1.7 (Alpha) during the second UK wave (n = 22) (*24*). At 83-84 weeks, 62 HCW had been recruited that were either SARS-CoV-2 infection naïve (n = 25) or had been infected during the first UK Wuhan, Hu-1 (n = 18), second B.1.1.7 (Alpha, n = 13) or third B.1.617.2 (Delta, n = 6) UK infection waves (table S1). All 62 had received a third dose of BNT162b2 at a median timepoint of 18 days (12, IQR) previously. Thirty-two HCW were recruited at 94-96 weeks, median 14 weeks (3w, IQR) after 3rd dose vaccination after the onset of the UK B.1.1.529 (Omicron) wave (table S6). This comprised n = 17 HCW with PCR-confirmed infection during the Omicron wave. Eleven of these were previously infection-naïve and 6 had prior Wuhan Hu-1 infection. A contemporaneous control group of HCW not infected during the Omicron wave was also recruited; this comprised 11 infection-naïve HCW and 4 with prior Wuhan Hu-1 infection. Lack of infection was confirmed by longitudinal N serology status (table S7).

### Isolation of PBMC

Peripheral blood mononuclear cells (PBMC) were isolated from heparinized blood using Histopaque®-1077 Hybri-Max^TM^ (Sigma-Aldrich) density gradient centrifugation in SepMate^TM^ tubes (Stemcell) as previously described (*20*, *23*, *24*). Isolated PBMCs were cryopreserved in fetal calf serum containing 10% DMSO and stored in liquid nitrogen.

### Isolation of serum

Whole blood samples in SST vacutainers (VACUETTE® #455092) were clotted at room temperature for at least 1h and then centrifuged for 10 min at 800g. Serum was aliquoted and stored at −80°C for SARS-CoV-2 antibody detection.

### Anti-N and Anti-S1 serology

Anti-nucleocapsid and anti-spike antibody detection testing was conducted at (UK Health Security Agency) using the Roche cobas^®^ e801 analyser. Anti-nucleocapsid antibodies were detected using the qualitative Roche Elecsys^®^ anti-SARS-CoV-2 electrochemiluminescence immune analyzer (ECLIA) nucleocapsid assay (Roche ACOV2, Product code: 09203079190) while anti-RBD antibodies were detected using the quantitative Roche Elecsys^®^ anti-SARS-CoV-2 ECLIA spike assay (Roche ACOV2S, Product code: 09289275190). Assays were performed and calibrated as recommended by the manufacturer. Anti-N results are expressed as a cutoff index (COI) value based on the electrochemiluminescence signal of a two-point calibration, with results COI ≥ 1.0 classified as positive. Anti-spike results are expressed as units per milliliter (U/ml) similarly based on a two-point calibration and a reagent specific master curve, with a quantitative range of 0.4 to 2,500 U/ml. Samples with a value of ≥1.0 U/ml are interpreted as positive for spike antibodies, and samples exceeding >250 U/ml are automatically diluted by the analyzer.

### Recombinant proteins

Wuhan Hu-1 SARS-CoV-2 S1 spike protein (Z03485-1), and B.1.617.2 (Delta) SARS-CoV-2 S1 spike protein (T19R, G142D, del 156-157, R158G, L425R, T478K, D614G, P681R) (Z03612-1) were purchased from GenScript USA Inc. B.1.1.529 (Omicron) SARS-CoV-2 S1 spike (A67V, H69del, V70del, T95I, G142D, V143del, Y144del, Y145del, N211del, L212I, ins214EPE, G339D, S371L, S373P, S375F, K417N, N440K, G446S, S477N, T478K, E484A, Q493R, G496S, Q498R, N501Y, Y505H, T547K, D614G, H655Y) and RBD (G339D, S371L, S373P, S375F, K417N, N440K, G446S, S477N, T478K, E484A, Q493R, G496S, Q498R, N501Y, Y505H) proteins (REC32006 and REC32007 respectively) were purchased from the Native Antigen Company.

### Peptides

Spike mapped epitope pool (MEP) comprises a previously described pool of eighteen 12-20mer peptide epitopes (*20*, *23*, *24*) (table S5A). B.1.1.529 (Omicron) and Wuhan Hu-1 peptide pools are comprised of peptides with the B.1.1.529 (Omicron) amino acid mutations and deletions and the respective Wuhan Hu-1 sequences (table S5B). They contain the predicted HLAII binding motifs as determined by NetMHCIIpan4.0 (*34*) (table S9). Peptides were synthesized by GL Biochem Shanghai Ltd (China).

### T cell assay by IFNγ-ELISpot

IFNγ-ELISpots were conducted as previously described (*20*, *23*, *24*). Precoated ELISpot plates (Mabtech 3420-2APT) were washed x4 with PBS, blocked for 1h (room temperature) with supplemented RPMI1640 (GibcoBRL) [10% heat inactivated FCS; 1% 100xpenicillin, streptomycin and L-Glutamine solutions (GibcoBRL)]. 200,000 PBMC were seeded/well and stimulated 18-22h at 37°C with SARS-CoV-2 recombinant protein [Wuhan Hu-1, B.1.617.2 (Delta) or B.1.1.529 (Omicron) SARS-CoV-2 S1 spike proteins (10 μg/ml)] or peptide pools (10 μg/ml/peptide). Negative and positive plate controls were medium or anti-CD3 (Mabtech mAb CD3-2). ELISpot plates were developed with 1 μg/ml biotinylated anti-human IFNγ detection Ab conjugated to alk-phosphatase (7-B6-1-ALP, Mabtech), diluted in PBS/0.5% FCS, adding 50 μl/well for 2h at room temperature followed by 50 μl/well BCIP/NBT-plus phosphatase substrate (Mabtech), 5 min (room temperature). Plates were washed and dried before analysis on an AID classic ELISpot plate reader (Autoimmun Diagnostika GMBH, Germany). ELISpot data was analyzed in Microsoft Excel. The average of two culture media alone wells was subtracted from all protein/peptide stimulated wells and any response that was lower in magnitude than 2 standard deviations of the sample specific control wells was not considered a stimulation-specific response. Results were expressed as difference in (delta) spot forming cells (SFC)/10^6^ PBMC between negative control and protein/peptide stimulation conditions. Results were excluded if negative control wells showed >100 SFC/10^6^ PBMC (n = 4) or cell viability was low with <1000 SFC/10^6^ PBMC in anti-CD3 positive control wells (n = 5). Results were plotted using Prism 9.0 for Mac OS (GraphPad).

### B cell ELISpots

Prior to B cell ELISpot assays PBMCs were cultured for 5d (37°C/5% CO_2_) in 24-well plates, 500,000 cells/well containing 1 μg/ml TLR7/8 agonist R848 plus 10 ng/ml recombinant human IL-2 (Mabtech Human Memory B cell Stimpack 3660-1). After 4d PBMC stimulation ELISpot PVDF plates (Millipore MSIPS4W10) were coated with PBS, purified anti-human IgG MT91/145 (10 μg/ml, Mabtech 3850-3-250), Wuhan Hu-1, B.1.617.2 or B.1.1.529 SARS-CoV-2 S1 spike proteins (10 μg/ml), and incubated at 4°C overnight. Plates were washed 5 times and blocked for 1h with RPMI1640 [supplemented with 10% heat inactivated FCS, 1% 100x penicillin, streptomycin and L-Glutamine solutions (GibcoBRL)]. Prestimulated PBMCs were washed twice before seeding at 15,000-7,500 cells/well for anti-human IgG coated wells and 150,000-15,000 cells/well for SARS-CoV-2 spike coated wells. Assays were run in duplicate. Plates were incubated, 37°C for 18-20h. For ELISpot development, plates were washed 5 times with PBS/0.05% Tween 20 (PBST) before incubation with 100 μl biotinylated anti-human IgG MT78/145 (Mabtech 3850-6-250), in PBS/0.5% FCS, 2h, room temperature. Plates were washed 5 times in PBST and incubated with 100 μl/well 1:1000 Streptavidin-ALP (Mabtech 3310-10-1000), in PBS/0.5% FCS for 1h, room temperature. Plates were then washed 5 times with PBST and spots developed by adding 100 μl/well BCIP/NBT substrate (Mabtech). Reactions were stopped by washing in tap water and plates dried before analyzing on an AID classic ELISpot plate reader (Autoimmun Diagnostika GMBH, Germany). Analysis of ELISpot data was performed in Microsoft Excel. Spots counted for each well were adjusted for cell numbers seeded and the average of PBS only coated wells subtracted from antigen coated wells. Number of SARS-CoV-2 spike antigen specific Ab secreting cells (ASC) was expressed as % of the total number of IgG ASC.

### B.1.1.529 (Omicron) RBD ELISA

Nunc 96-well immune ELISA plates were coated with 1 μg/ml of B.1.1.529 (Omicron) Spike RBD protein in carbonate buffer (Sigma Aldrich) for 2 hours at 37°C before washing with PBS (0.05% Tween) (PBST) and blocking at 37°C for 1 hour with PBS containing 1% Bovine Serum Albumin (BSA). Plates were washed in PBST again before application of 50 μl of diluted sera to each well. All serum dilutions were run in duplicate and a four-point dilution series was run for each sample. Following overnight incubation at 4°C, plates were washed with PBST and wells incubated with 1:1000 dilution of Biotin Mouse Anti-human IgG (BD Pharmingen, 555785) at room temperature for 1 hour. Plates were washed again before application of 1:200 dilution of Streptavidin Horseradish Peroxidase (HRP) (Bio-techne, DY998) for 30 min followed by a final wash and then assay development using 3,3′, 5,5;-tetramethylbenzidine (TMB) substrate (Sigma Aldrich, T0440). Color development was stopped after 5 min by the addition of 0.18M H_2_SO_4_ and OD450nm values for each well measured using a FLUOstar® Omega Plate Reader. Analysis of ELISA data was performed in Prism 9.0 for Mac OS (GraphPad). Data for serial dilutions were plotted and area under the curve calculated for each individual serum sample.

### Multiplex variant-specific IgG antibody measurement

Antibody titers against VOC-specific spike antigens (RBD or spike) were measured using the multiplex MesoScale Discovery (MSD) electro-chemiluminescent immunoassay (V-Plex, MSD, Gaithersburg). IgG binding antibody to the RBD domain for the different VOC were determined using the V-Plex Panel 22 (Catalogue number K15559U) which includes the RBD antigens of “WT” SARS-CoV-2, B.1.1.529/BA.1 (Omicron), B.1.1.7 (Alpha), B.1.351/B.1.351.1 (Beta), P.1 (Gamma) and AY.3/AY.4/B.1.617.2 (Delta). IgG binding antibody to the full spike protein of the different VOC were determined using V-Plex Panel 23 (Catalogue numb er K15567U) which includes spike antigens of “WT” SARS-CoV-2, AY.4.2 (Delta sub-lineage), B.1.1.529/BA.1 (Omicron), B.1.1.7 (Alpha), B.1.351 (Beta), P.1 (Gamma), B.1.617.2/AY.3/ AY.5 (Delta and Delta sub-lineages) and AY.4 (Delta alternative sequence and sub-lineages) (*36*, *37*) A full list of each antigen and their included mutations can be found in the supplementary material (table S2).

In brief, plates were run as per manufacturer’s instructions, with washing between incubations performed using a Biotek 405TS plate washer. Plates were blocked for 30 min with 5% BSA before the addition of samples diluted between 1:1,000 to 1:100,000. Samples were incubated for 2 hours, followed by addition of the secondary anti-human IgG antibody (Sulfo-tag) for 1 hour. Read buffer was added to plates before reading immediately using the MSD QuickPlex SQ 120 platform. Only results from antigen spots within the detection range were used for the final analysis. Results were reported as arbitrary units per milliliter (AU/ml) determined against a 7-point calibration curve using serially diluted reference standard 1.

### Authentic Wuhan Hu-1 SARS-CoV-2 and VOC variant titration

SARS-CoV-2 isolate stocks [including Wuhan Hu-1, B.1.1.7 (Alpha), B.1.351 (Beta), P.1 (Gamma), B.1.617.2 (Delta) and B.1.1.529 (Omicron)] used in experiments (table S8) were prepared and titrated as previously described (*23*, *24*).

### Authentic Wuhan Hu-1 SARS-CoV-2 and VOC microneutralization assays

SARS-CoV-2 microneutralization assays were carried out as described previously (*23*, *24*). VeroE6 cells were seeded in 96-well plates 24h prior to infection. Duplicate titrations of heat-inactivated participant sera were incubated with 3x10^4^ FFU SARS-CoV-2 virus (TCID100) at 37°C, 1h. Serum/virus preparations were added to cells and incubated for 72h. Surviving cells were fixed in formaldehyde and stained with 0.1% (wt/vol) crystal violet solution (crystal violet was resolubilized in 1% (wt/vol) sodium dodecyl sulfate solution). Absorbance readings were taken at 570nm using a CLARIOStar Plate Reader (BMG Labtech). Negative controls of pooled pre-pandemic sera (collected before 2008), and pooled serum from neutralization positive SARS-CoV-2 convalescent individuals were spaced across the plates. Absorbance for each well was standardized against technical positive (virus control) and negative (cells only) controls on each plate to determine percentage neutralization values. IC50s were determined from neutralization curves. All authentic SARS-CoV-2 propagation and microneutralization assays were performed in a containment level 3 facility.

### In silico epitope prediction for B.1.1.529 (Omicron) and BA.2

In silico predictions of HLA-DRB1 peptide-binding were performed using NetMHCIIpan-4.0 (*38*) based on peptide length of 15 amino acids (tables S9 and S13). HLA core binding sequences containing individual mutations were selected if within a peptide defined as a strong or weak binder by the NetMHCIIpan-4.0 default parameters of rank score <1% (threshold for strong binder) and rank score <5% (threshold for weak binder). For HLA-A, HLA-B and HLA-C alleles, analysis was performed using NetMHCpan-4.1 based on peptide lengths of 8, 9 and 10 amino acids (tables S10 to S12 and S14 to S16). Again the default parameters of rank score ≤ 0.5% (threshold for strong binder) and ≤ 2% (threshold for weak binder) were used.

### HLA-DRB1*0401 transgenic T cell assays

Studies using HLAII transgenics carrying DRB1*0401 in the context of a homozygous knockout for murine H2-Aβ have been previously described (*39*, *40*). Mice (7-8 weeks) were immunized in one hind footpad with B.1.1.529 (Omicron) variant or Wuhan Hu-1 pools/peptides containing 10 μg each peptide sequence in Hunters Titermax Gold adjuvant (Sigma Aldrich). Popliteal lymph nodes were collected at d10 and prepared as single cell suspensions. IFNγ ELISpot assays were performed in triplicate in HL1 serum-free medium (Lonza) [supplemented with 1% 100x L-glutamine and 0.5% 100x penicillin/streptomycin solutions (GibcoBRL)]. PVDF ELISpot plates (Merck Millipore MSIPN4550) were coated with anti-mouse IFNγ capture antibody (Diaclone Murine IFN gamma ELISpot Set, 862.031.020) overnight before seeding 200,000 lymph node cells/well and stimulating (72h, 37°C with 5% CO_2_) with peptide pools or individual SARS-CoV-2 Wuhan Hu-1 or variant peptides (10 μg/ml/peptide). Internal plate controls were culture media alone and staphylococcal enterotoxin B (SEB). Assays were developed using biotinylated anti-mouse IFNγ followed by streptavidin-alkaline phosphatase conjugate and BCIP/NTB substrate (Diaclone) before washing in tap water, drying and analyzing using an AID classic ELISpot plate reader (Autoimmun Diagnostika GMBH, Germany). Analysis of ELISpot data was performed in Microsoft Excel. The average from 3 culture media wells was subtracted from peptide-stimulated wells and any response that was <2SD of the sample specific control wells was not considered a peptide-specific response. Results were expressed as difference in (delta) SFC/10^6^ PBMC between the negative control and peptide stimulation conditions. Results were plotted using Prism 9.0 for Mac OS (GraphPad).

For transcriptomic analysis, lymph node cells were cultured with no peptide, or 10 μg/ml Wuhan Hu-1 or B.1.1.529 (Omicron) variant G142/del143-5 or Q493R/G496S/Q498R/N501Y/Y505H peptides. At 24h, cells were harvested and lysed for RNA extraction. RNA was extracted using an Agilent RNA microprep kit. cDNA was prepared using an RT2 first strand kit (Qiagen) and qPCR for target genes performed using RT^2^ profiler PCR array Mouse T Helper Cell Differentiation plates (Qiagen PAMM-503Z). Data were analyzed and plotted using the Qiagen GeneGlobe data analysis tool and genes up-regulated by peptide stimulation with a p value > 0.05 (by students *t* test) compared to no peptide stimulation were identified.

### Statistics and reproducibility

Data was assumed to have a non-Gaussian distribution. Wilcoxon matched-pairs signed rank test and a Mann-Whitney U-test were used for single, paired and unpaired comparisons. Non-parametric tests were used throughout. P value of <0.05 was considered significant. Prism 9.0 for Mac was used for analysis.

## Supplementary Materials

### This PDF file includes:

Figs. S1 to S6 Tables S1 to S17 COVIDsortium Investigators COVIDsortium Immune Correlates Network

### Other Supplementary Material for this manuscript includes the following:

MDAR Reproducibility Checklist
