# Supplementary material for: Immune boosting by B.1.1.529 (Omicron) depends on previous SARS-CoV-2 exposure
Source: Science. 2022 Jun 14:eabq1841. doi: 10.1126/science.abq1841 (PMC9210451; doi:10.1126/science.abq1841)
Supplement: Supplementary file 1 — Figs. S1 to S6 Tables S1 to S17 COVIDsortium Investigators COVIDsortium Immune Correlates Network [file science.abq1841_sm.pdf]

Science
AAAS

Supplementary Materials for

Immune boosting by B.1.1.529 (Omicron) depends on previous SARS-CoV-2 exposure

Catherine J. Reynolds *et al.*

Corresponding author: Rosemary J. Boyton, r.boyton@imperial.ac.uk

DOI: 10.1126/science.abq1841

The PDF file includes:

Figs. S1 to S6
Tables S1 to S16
COVIDsortium Investigators
COVIDsortium Immune Correlates Network

Other Supplementary Material for this manuscript includes the following:

MDAR Reproducibility Checklist
Table S17

This PDF file includes:

Fig. S1. CONSORT diagram of UK COVIDsortium HCW cohort and vaccine sub-study cohorts.

Fig. S2. Antibody binding responses against Wuhan Hu-1 or VOC whole spike in BNT162b2 triple-vaccinated HCW that were infection-naïve or infected during the Wuhan Hu-1, B.1.1.7 (Alpha) or B.1.617.2 (Delta) SARS-CoV-2 waves.

Fig. S3. S1 RBD and whole spike VOC antibody binding correlation plots with authentic live virus cross-neutralization nAb IC50 against VOC in triple-vaccinated HCW at two to three weeks after third BNT162b2 vaccine dose.

Fig. S4. nAb IC50 against B.1.1.529 (Omicron) live virus and T cell responses against B.1.1.529 (Omicron) S1 protein in triple-vaccinated HCW at two to three weeks after the third BNT162b2 vaccine dose.

Fig. S5. nAb IC50 against SARS-CoV-2 VOC including B.1.1.529 (Omicron) were not boosted by infection during the B.1.1.529 (Omicron) wave in HCW that had been previously infected during the Wuhan Hu-1 wave.

Fig. S6. RBD and whole spike VOC antibody binding correlation plots with authentic live virus cross-neutralization nAb IC50 against VOC in triple-vaccinated HCW at a median of 14 weeks after third BNT162b2 vaccine dose.

Table S1. HCW recruited at two to three weeks after the third BNT162b2 vaccine dose: COVID-19 vaccine sub-study HCW cohort with laboratory confirmed SARS-CoV-2 infection during the first Wuhan Hu-1, second B.1.1.7 (Alpha), and third B.1.617.2 (Delta) UK waves and infection naïve HCW.

Table S2. Mutations present in SARS-CoV2 wild-type and VOC antigens used in multiplex MesoScale Discovery (MSD) assays

Table S3. Cross-reactive S1 RBD IgG antibody binding and nAb IC50 against B.1.1.529 (Omicron) reduced compared to other VOC in triple-vaccinated HCW

Table S4. MBC frequency against ancestral Wuhan Hu-1 and B.1.617.2 (Delta) S1 were similar, but significantly reduced against B.1.1.529 (Omicron) S1 at 21-21 weeks after the second vaccine dose and 2-3 weeks after the third vaccine dose.

Table S5A. Spike mapped epitope peptide (MEP) pool.

Table S5B. B.1.1.529 (Omicron) peptide pool and the matched sequence peptide pool for ancestral Wuhan Hu-1.

Table S6. HCW recruited at a median of 14 weeks after the third BNT162b2 vaccine dose with laboratory confirmed SARS-CoV-2 infection during the first Wuhan Hu-1 and fourth B.1.1.529 (Omicron) UK waves and infection naïve HCW.

Table S7. HCW recruited at a median of 14 weeks after the third BNT162b2 vaccine dose during the B.1.1.529 (Omicron) wave.

Table S8. Spike mutations in VOC viral isolates used in this study.

Table S9. *In silico* predictions of B.1.1.529 (Omicron) variant peptide binding to common UK HLAII alleles.

Table S10. *In silico* predictions of B.1.1.529 (Omicron) variant peptide binding to common UK HLA-A alleles.

Table S11. *In silico* predictions of B.1.1.529 (Omicron) variant peptide binding to common UK HLA-B alleles.

Table S12. *In silico* predictions of B.1.1.529 (Omicron) variant peptide binding to common UK HLA-C alleles.

Table S13. *In silico* predictions of BA.2 variant peptide binding to common UK HLAII alleles.

Table S14. *In silico* predictions of BA.2 variant peptide binding to common UK HLA-A alleles.

Table S15. *In silico* predictions of BA.2 variant peptide binding to common UK HLA-B alleles.

Table S16. *In silico* predictions of BA.2 variant peptide binding to common UK HLA-C alleles.

UK COVIDsortium Investigators.

UK COVIDsortium Immune Correlates Network.

Table S17. Raw data file (.xlsx).

S1. CONSORT diagram of UK COVIDsortium HCW cohort. CONSORT flow diagram showing recruitment into parent longitudinal COVIDsortium London HCW cohort and vaccine sub-study cohorts.

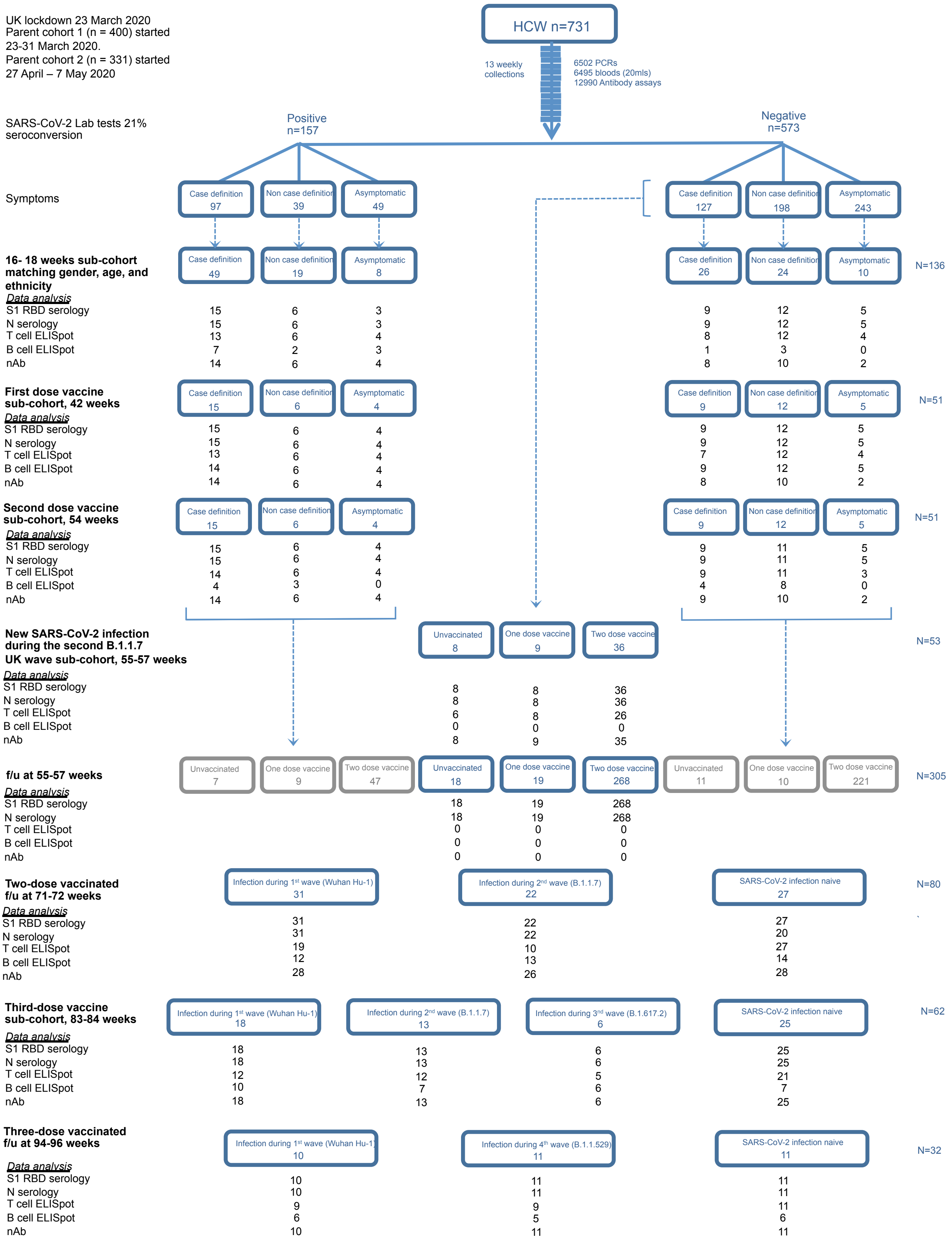

Fig. S2. Antibody binding responses against Wuhan Hu-1 or VOC whole spike in BNT162b2 triple-vaccinated HCW that were infection-naïve or infected during the Wuhan Hu-1, B.1.1.7 (Alpha) or B.1.617.2 (Delta) SARS-CoV-2 waves. Serum Ab binding against Wuhan Hu-1, B.1.1.7 (Alpha), B.1.351 (Beta), P.1 (Gamma), B.1.617.2 (Delta) or B.1.1.529 (Omicron) whole spike protein 2-3w after the third BNT162b2 dose in infection-naïve HCW (blue, n = 25) or HCW with lab-confirmed SARS-CoV-2 infection during the ancestral Wuhan Hu-1 (red, n = 18), B.1.1.7 (Alpha, green, n = 13) or B.1.617.2 (Delta, purple, n = 6) waves. Statistics were calculated using Prism 9.0. Mann-Whitney U test. Ab, antibody; AU, arbitrary units; HCW, health care workers; VOC, variant of concern; w, weeks.

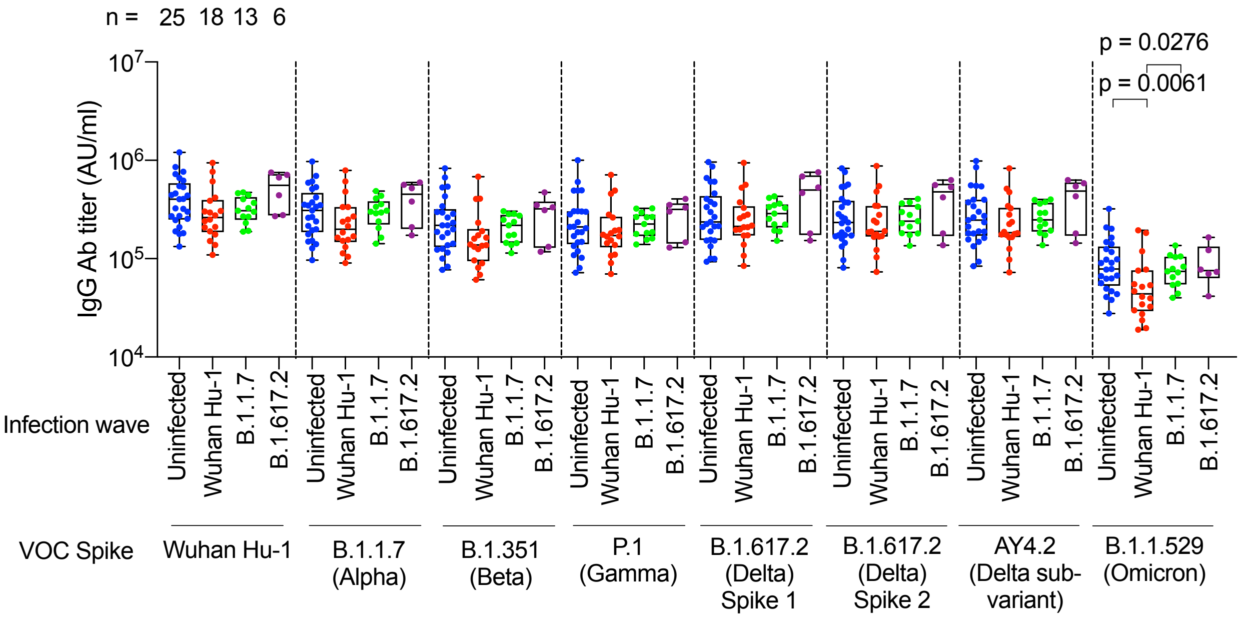

Fig. S3. S1 RBD and whole spike VOC antibody binding correlation plots with authentic live virus cross-neutralization nAb IC50 against VOC in triple-vaccinated HCW at two to three weeks after third BNT162b2 vaccine dose. Correlations between (**A**) Whole spike and S1 RBD IgG Ab, (**B**) nAb IC50 and S1 RBD IgG (**C**) nAb IC50 and whole spike IgG for ancestral Wuhan Hu-1, B.1.1.7 (Alpha), B.1.351(Beta), P.1 (Gamma), B.1.617.2 (Delta) and B.1.1.529 (Omicron) VOC 2-3w after third vaccine dose. Data shown are for infection-naïve HCW (blue, n = 25) and HCW with laboratory confirmed SARS-CoV-2 infection during the ancestral Wuhan Hu-1 (red, n = 18), B.1.1.7 (Alpha, green, n = 13) and B.1.617.2 (Delta, purple, n = 6) waves. Statistical tests were performed using Prism 9.0. (**A, B, C**) Spearman's rank correlation. Ab, antibody; AU; arbitrary units; HCW, health care workers; nAb; neutralizing antibody; RBD, receptor binding domain; S1, subunit 1; VOC, variant of concern.

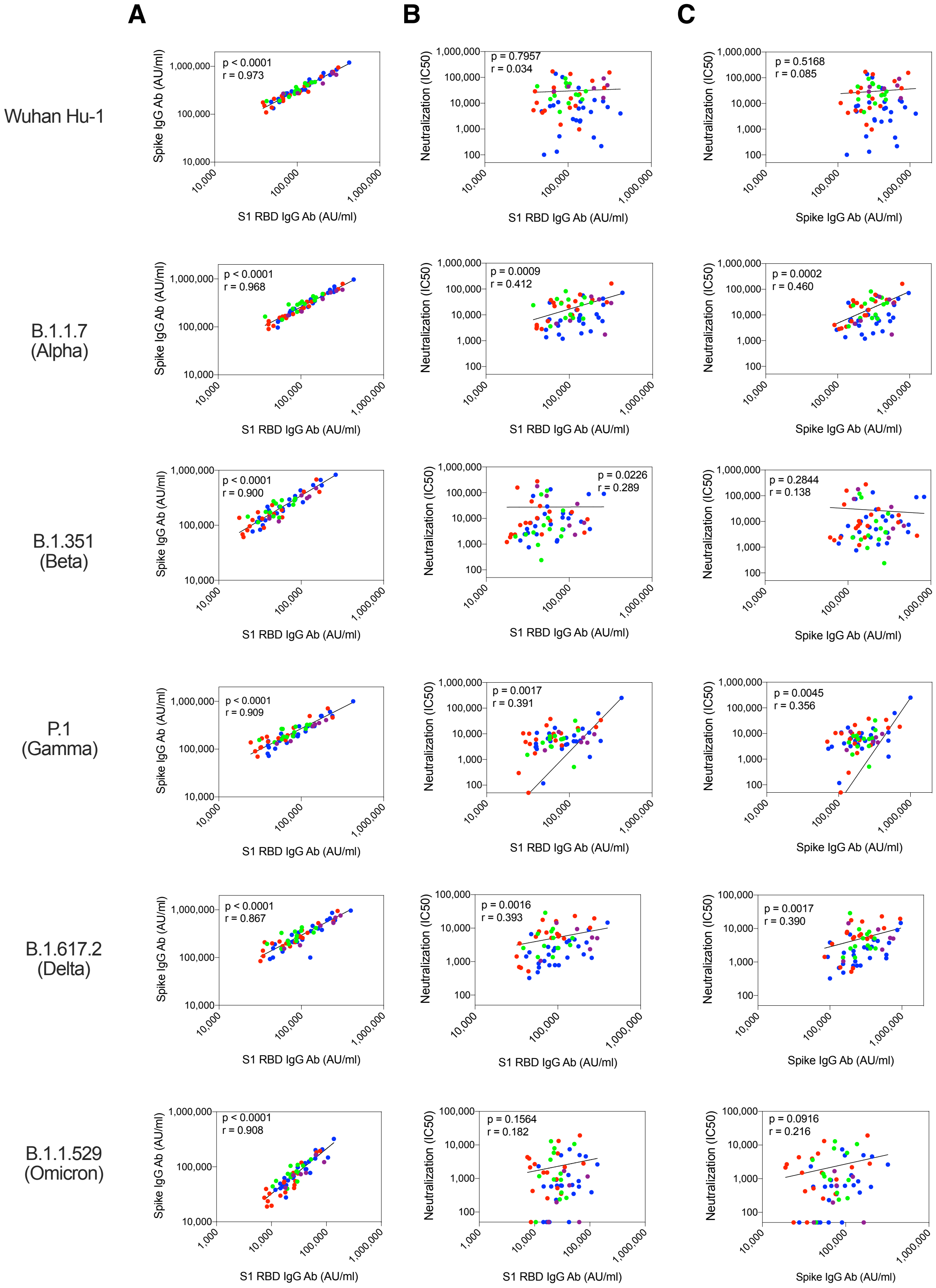

Fig. S4. nAb IC50 against B.1.1.529 (Omicron) live virus and T cell responses against B.1.1.529 (Omicron) S1 protein in triple-vaccinated HCW at two to three weeks after the third BNT162b2 vaccine dose. Correlation plot of T cell response against B.1.1.529 (Omicron) S1 protein and nAb IC50 against B.1.1.529 (Omicron) live virus in infection-naïve HCW (blue, n = 25) and HCW with laboratory confirmed SARS-CoV-2 infection during the Wuhan Hu-1 (red, n = 18), B.1.1.7 (Alpha, green, n = 13) and B.1.617.2 (Delta, purple, n = 6) waves at 2-3w after the third vaccine dose. Statistical tests were performed using Prism 9.0. Spearman's rank correlation. HCW, health care workers; PBMC, peripheral blood mononuclear cells; nAb, neutralizing antibody, S1, subunit 1; SFC, spot forming cells; w, weeks.

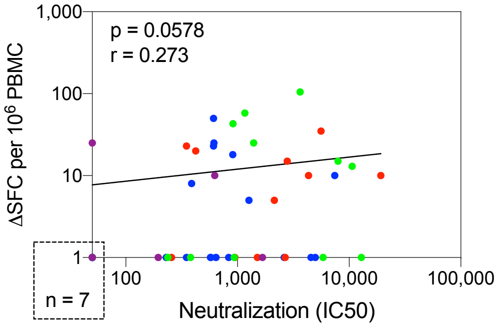

Fig. S5. nAb IC50 against SARS-CoV-2 VOC including B.1.1.529 (Omicron) were not boosted by infection during the B.1.1.529 (Omicron) wave in HCW that had been previously infected during the Wuhan Hu-1 wave. Change in nAb IC50 against Wuhan Hu-1, B.1.1.7 (Alpha), B.1.351 (Beta), P.1 (Gamma), B.1.617.2 (Delta) or B.1.1.529 (Omicron) live virus between 2-3w and 14w (median 14, IQR 3) after third vaccine dose in (**A)** paired analysis of infection-naïve HCW (blue, n = 9) not infected during the B.1.1.529 (Omicron) wave and (**B**) paired analysis of HCW with laboratory confirmed SARS-CoV-2 infection during the Wuhan Hu-1 wave in March 2020 (red, n = 8). Of those HCW infected during the Wuhan Hu-1 wave, a subset was re-infected during the B.1.1.529 (Omicron) wave (red open triangles, n = 4). Statistical tests were performed using Prism 9.0. (**A**, **B)** Wilcoxon matched-pairs signed rank test. HCW, health care workers; IQR, inter-quartile range; nAb, neutralizing antibody; VOC, variant of concern.

A

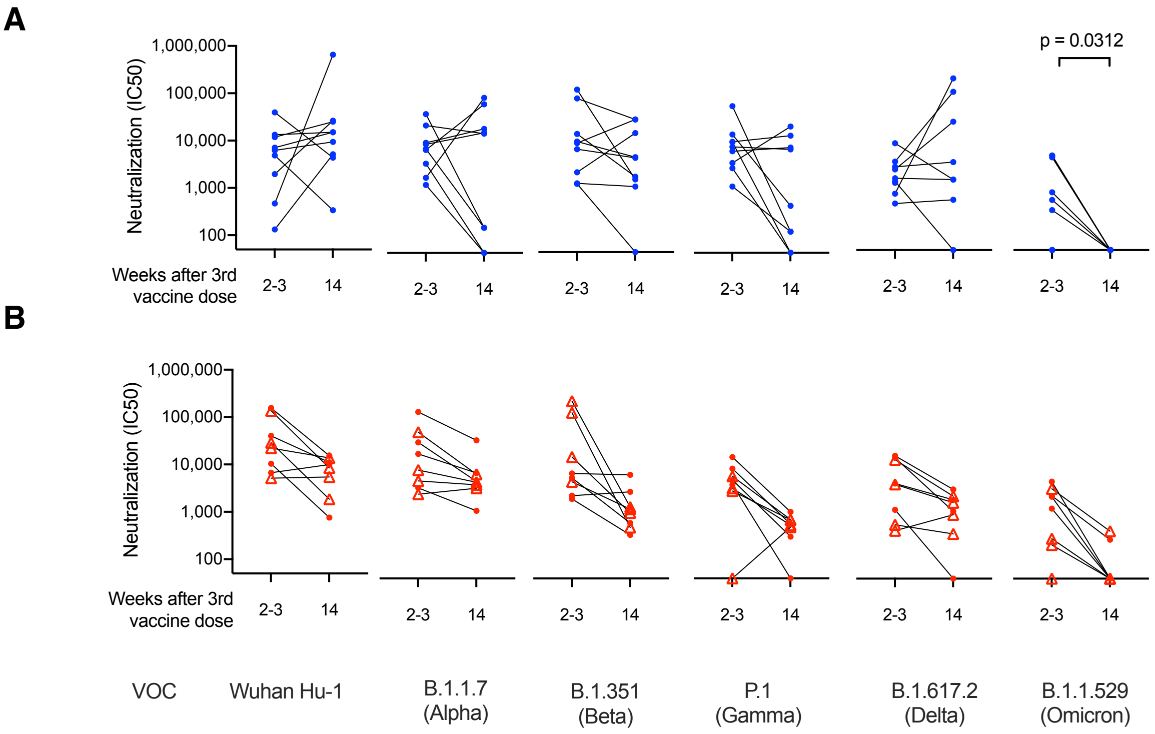

Fig. S6. S1 RBD and whole spike VOC antibody binding correlation plots with authentic live virus cross-neutralization nAb IC50 against VOC in triple-vaccinated HCW at a median of 14 weeks after third BNT162b2 vaccine dose. Correlations between (**A**) Whole spike and S1 RBD IgG Ab, (**B**) nAb IC50 and S1 RBD IgG (**C**) nAb IC50 and whole spike IgG for ancestral Wuhan Hu-1, B.1.1.7 (Alpha), B.1.351 (Beta), P.1 (Gamma), B.1.617.2 (Delta) and B.1.1.529 (Omicron) VOC 14 w (median 14w, IQR 3w) after third vaccine dose. Data shown are for infection naïve HCW (blue, n = 11) and HCW with laboratory confirmed SARS-CoV-2 infection during the Wuhan Hu-1 (red, n = 4), B.1.1.529 (Omicron, black, n = 11) and Wuhan Hu-1 followed by B.1.1.529 (Omicron, pink, n = 6) waves. Statistical tests were performed using Prism 9.0. (**A**, **B**, **C**) Spearman's rank correlation. Ab, antibody; AU; arbitrary units; HCW, health care workers; IQR, inter-quartile range; nAb, neutralizing antibody; RBD, receptor binding domain; S1, subunit 1; VOC, variant of concern; w, weeks.

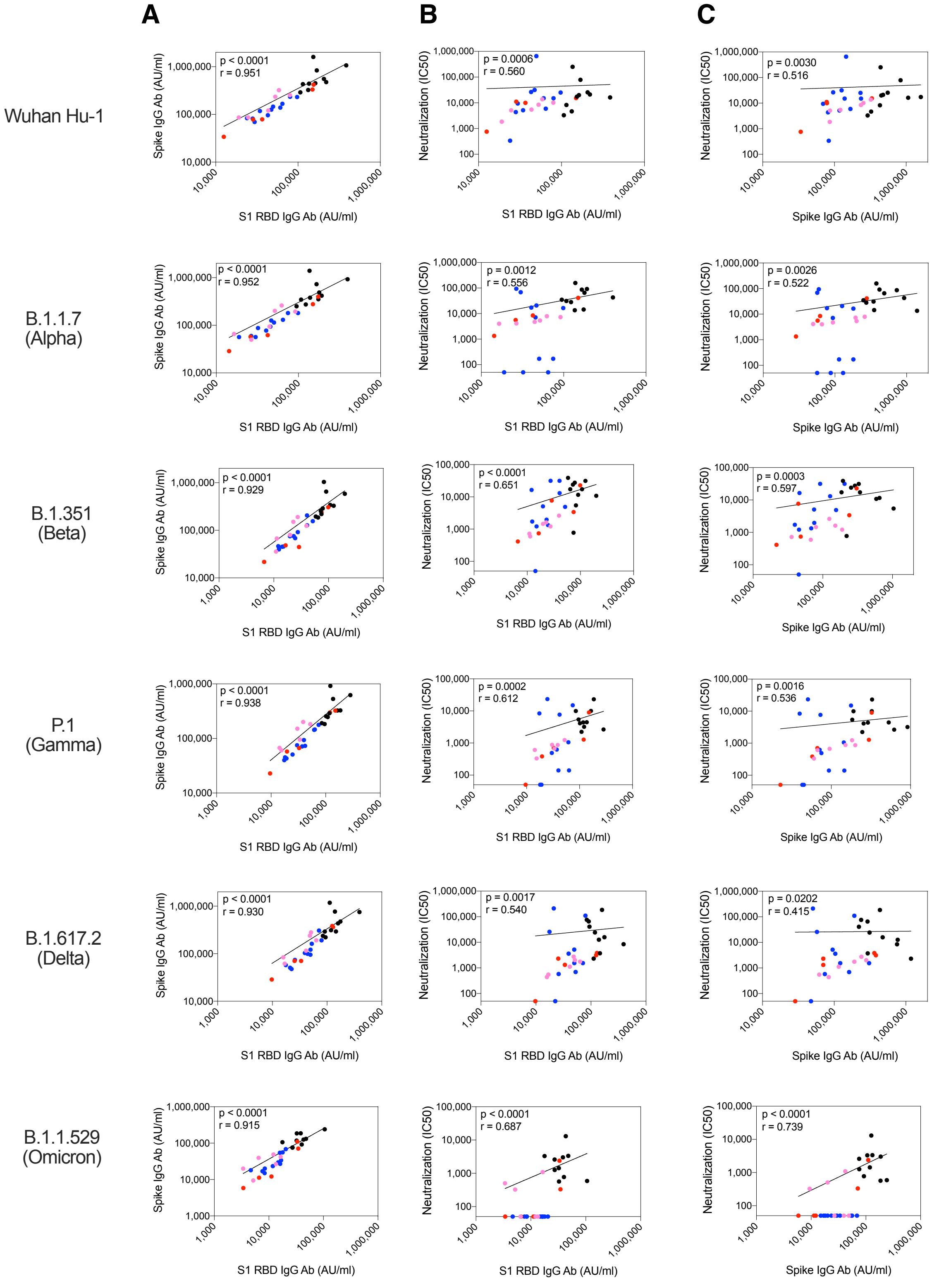

Table S1. HCW recruited at two to three weeks after the third BNT162b2 vaccine dose: COVID-19 vaccine sub-study HCW cohort with laboratory confirmed SARS-CoV-2 infection during the first Wuhan Hu-1, second B.1.1.7 (Alpha), and third B.1.617.2 (Delta) UK waves and infection naïve HCW.

	Total	SARS-CoV-2 infection naïve<br><br>SARS-CoV-2 PCR negative<br><br>Roche S1-RBD Ab and N Ab negative	SARS-CoV-2 infection during the first UK Wuhan Hu-1 wave	SARS-CoV-2 infection during the second UK B.1.1.7 (Alpha) wave	SARS-CoV-2 infection during the third UK B.1.617.2 (Delta) wave
HCW<br><br>n (% of total)	62 (100)	25 (40)	18 (29)	13 (21)	6 (10)
Mean age (range)	41 (25-60)	43 (25-60)	42 (26-58)	40 (25-57)	39 (27-55)
Gender:					
Female n (%)	37 (60)	17 (68)	7 (39)	8 (62)	5 (83)
Ethnicity:					
White n (%)	46 (74)	18 (72)	14 (78)	9 (69)	5 (83)
Minority ethnic group (UK) n (%)	16 (26)	7 (28)	4 (22)	4 (31)	1 (17)
BNT162b2 COVID-19 vaccine, n (%)	62 (100)	25 (100)	18 (100)	13 (100)	6 (100)
Number of days since third vaccine dose Median (IQR)	18 (10)	17 (9)	19 (11)	15 (12)	14 (5)

Abbreviations: Ab, antibody; HCW, healthcare worker; IQR, inter-quartile range; N, nucleocapsid; RBD, receptor binding domain; S1, subunit 1

Table S2. Mutations present in SARS-CoV2 wild-type and VOC antigens used in multiplex MesoScale Discovery (MSD) assays

**Spike RBD domain**	
**WT -** SARS-CoV-2 S1 RBD	R319-F541 of the SARS-CoV-2 Spike Sequence; C-terminal His-Tag
**Delta -** AY.3, AY.4, AY.4.2, AY.5, AY.6, AY.7, AY.12, AY.14, B.1.617.2, B.1.617.2+ΔY144	R319-F541 of the SARS-CoV-2 Spike Sequence; C-terminal His-Tag; L452R, T478K
**Omicron -** B.1.1.529; BA.1	R319-F541 of the SARS-CoV-2 Spike Sequence; C-terminal His-Tag; G339D, S371L, S373P, S375F, K417N, N440K, G446S, S477N, T478K, E484A, Q493R, G496S, Q498R, N501Y, Y505H
**Beta -** B.1.351; B.1.351.1	R319-F541 of the SARS-CoV-2 Spike Sequence; C-terminal His-Tag; K417N, E484K, N501Y
**Gamma -** P.1	R319-F541 of the SARS-CoV-2 Spike Sequence; C-terminal His-Tag; K417T, E484K, N501Y
**Alpha -** B.1.1.7	R319-F541 of the SARS-CoV-2 Spike Sequence; C-terminal His-Tag; N501Y
**Full spike**	
**WT -** SARS-CoV-2 Spike	Soluble ectodomain with T4 trimerization domain; C-terminal Strep-Tag and His-Tag
**Omicron -** B.1.1.529; BA.1	Soluble ectodomain with T4 trimerization domain; C-terminal Strep-Tag and His-Tag; A67V, ΔH69-V70, T95I, G142D, Δ143-145, Δ211/L212I, ins214EPE, G339D, S371L, S373P, S375F, K417N, N440K, G446S, S477N, T478K, E484A, Q493R, G496S, Q498R, N501Y, Y505H, T547K, D614G, H655Y, N679K, P681H, N764K, D796Y, N856K, Q954H, N969K, L981F
**Delta sub-lineage -** AY.4.2	Soluble ectodomain with T4 trimerization domain; C-terminal Strep-Tag and His-Tag; T19R, T95I, G142D, Y145H, Δ156/157, R158G, A222V L452R, T478K, D614G, P681R, D950N
**Delta -** B.1.617.2; AY.4 (Alt Seq 2)	Soluble ectodomain with T4 trimerization domain; C-terminal Strep-Tag and His-Tag; (Alt Seq 2): T19R, T95I, G142D, Δ156/157, R158G, L452R, T478K, D614G, P681R, D950N
**Gamma -** P.1	Soluble ectodomain with T4 trimerization domain; C-terminal Strep-Tag and His-Tag; L18F, T20N, P26S, D138Y, R190S, K417T, E484K, N501Y, D614G, H655Y, T1027I, V1176F
**Alpha -** B.1.1.7	Soluble ectodomain with T4 trimerization domain; C-terminal Strep-Tag and His-Tag; Δ69-70, Δ144, N501Y, A570D, D614G, P681H, T716I, S982A, D1118H
**Beta -** B.1.351	Soluble ectodomain with T4 trimerization domain; C-terminal Strep-Tag and His-Tag; L18F, D80A, D215G, Δ242-244, R246I, K417N, E484K, N501Y, D614G, A701V
B.1.617.2; AY.3; AY.5; AY.6; AY.7; AY.14 - Alt Seq 1	Soluble ectodomain with T4 trimerization domain; C-terminal Strep-Tag and His-Tag; (Alt Seq 1): T19R, G142D, Δ156/157, R158G, L452R, T478K, D614G, P681R, D950N

Table S3. Cross-reactive S1 RBD IgG antibody binding and nAb IC50 against B.1.1.529 (Omicron) reduced compared to other VOC in triple-vaccinated HCW

		VOC					
		Ancestral Wuhan Hu-1	B.1.1.7 (Alpha)	B.1.351 (Beta)	P.1 (Gamma)	B.1.617.2 (Delta)	B.1.1.529 (Omicron)
**S1 RBD IgG antibody titer (AU/ml)**<br>Geo mean, **fold difference**, p value [VOC compared to ancestral Wuhan Hu-1]							
Infection history	Infection naïve (n=25)	120985	118842<br>↓1.02<br>p=0.2752	62139<br>**↓1.95**<br>p<0.0001	89709<br>**↓1.35**<br>p<0.0001	101524<br>**↓1.19**<br>p<0.0001	33374<br>**↓3.63**<br>p<0.0001
	Wuhan Hu-1 (n=18)	91120	89474<br>↓1.02<br>p=0.1964	41015<br>**↓2.22**<br>p<0.0001	56658<br>**↓1.61**<br>p<0.0001	77322<br>**↓1.18**<br>p<0.0001	18625<br>**↓4.89**<br>p<0.0001
	B.1.1.7 (Alpha) (n=13)	95051	96750<br>↑1.02<br>p=0.3757	49515<br>**↓1.92**<br>p=0.0002	70929<br>**↓1.34**<br>p=0.0002	81735<br>**↓1.16**<br>p=0.0002	23967<br>**↓3.97**<br>p=0.0002
	B.1.617.2 (Delta) (n=6)	175818	171560<br>↓1.02<br>p>0.9999	88926<br>**↓1.98**<br>p=0.0312	116731<br>**↓1.51**<br>p=0.0312	163872<br>**↓1.07**<br>p=0.0625	39551<br>**↓4.45**<br>p=0.0312
**nAb (IC50)**<br>Geo mean, **fold difference**, p value [VOC compared to ancestral Wuhan Hu-1]							
Infection history	Infection naïve (n=25)	4015	7175<br>↑1.79<br>p=0.1974	7827<br>↑1.95<br>p=0.3748	5854<br>↑1.46<br>p=0.9218	1862<br>**↓2.16**<br>p=0.0087	202<br>**↓19.88**<br>p<0.0001
	Wuhan Hu-1 (n=18)	16745	16257<br>↓1.03<br>p=0.5798	9383<br>↓1.78<br>p=0.3465	4579<br>**↓3.66**<br>p=0.0432	4921<br>**↓3.40**<br>p=0.0007	831<br>**↓20.15**<br>p<0.0001
	B.1.1.7 (Alpha) (n=13)	25068	19219<br>↓1.30<br>p=0.8394	4923<br>**↓5.09**<br>p=0.0266	5106<br>**↓4.91**<br>p=0.0024	4152<br>**↓6.04**<br>p=0.0002	979<br>**↓25.61**<br>p=0.0002
	B.1.617.2 (Delta) (n=6)	39025	11835<br>↓3.30<br>p=0.0625	18567<br>↓2.10<br>p=0.8438	5836<br>**↓6.69**<br>p=0.0312	4542<br>**↓8.59**<br>p=0.0312	78<br>**↓500.32**<br>p=0.0312

Abbreviations: AU, arbitrary units; nAb, neutralizing antibody; RBD, receptor binding domain; S1, Subunit 1; VOC, variant of concern. Statistical tests were performed using Prism 9.0. Wilcoxon matched-pairs signed rank test

Table S4. MBC frequency against ancestral Wuhan Hu-1 and B.1.617.2 (Delta) S1 were similar, but significantly reduced against B.1.1.529 (Omicron) S1 at 21-21 weeks after the second vaccine dose and 2-3 weeks after the third vaccine dose.

		S1 protein		
		Ancestral Wuhan Hu-1	B.1.617.2 (Delta)	B.1.1.529 (Omicron)
**20-21 weeks after second vaccine dose**<br>Geo mean % S1 specific IgG+ ASC, **fold difference**, p value, (compared to ancestral Wuhan Hu-1)				
Infection history	Infection naïve (n=9)	3.09	2.71<br>↓1.1<br>p=0.0742	1.23<br>**↓2.5**<br>p=0.0039
	Wuhan Hu-1 (n=9)	3.32	3.47<br>1.0<br>p=0.8867	1.52<br>**↓2.2**<br>p=0.0039
	B.1.1.7 (Alpha) (n=9)	8.02	9.15<br>↑1.1<br>p=0.4258	3.95<br>**↓2.0**<br>p=0.0039
	B.1.617.2 (Delta) (n=3)	3.97	3.75<br>↓1.1<br>p>0.9999	1.36<br>↓2.9<br>p=0.1250
**2-3 weeks after third vaccine dose**<br>Geo mean % S1 specific IgG+ ASC, **fold difference**, p value (compared to ancestral Wuhan Hu-1)				
Infection history	Infection naïve (n=7)	9.27	8.62<br>↓1.1<br>p=0.2969	4.61<br>**↓2.0**<br>p=0.0156
	Wuhan Hu-1 (n=10)	8.08	8.65<br>↑1.1<br>p=0.3750	3.33<br>**↓2.4**<br>p=0.0020
	B.1.1.7 (Alpha) (n=7)	17.27	18.56<br>↑1.1<br>p=0.0781	9.18<br>**↓1.9**<br>p=0.0312
	B.1.617.2 (Delta) (n=6)	9.79	9.30<br>↓1.1<br>p=0.4375	3.33<br>**↓2.9**<br>p=0.0312

Abbreviations: ASC, antibody secreting cells; MBC, memory B cell; S1, subunit 1. Statistical tests were performed using Prism 9.0. Wilcoxon matched-pairs signed rank test.

Table S5A. Spike mapped epitope peptide (MEP) pool.

Region	Position/mutation	Amino acid sequence	Peptide sequence with the site of B.1.1.529 (Omicron) mutations indicated in grey
Spike (S1)	166-180	CTFEYVSQPFLMDLE	
Spike (S1)	191-205	EFVFKNIDGYFKIYS	
Spike (S1)	206-220	KHTPINLVRDLPQGF	KHTPIIVREPEDLPQGF
Spike (S1)	211-225	NLVRDLPQGFSALEP	IVREPEDLPQGFSALEP
Spike (S1 RBD)	351-365	YAWNRKRISNCVADY	
Spike (S1 RBD)	381-395	GVSPTKLNDLCFTNV	
Spike (S1 RBD)	446-460	GGNYNYLYRLFRKSN	SGNYNYLYRLFRKSN
Spike (S1 RBD)	451-465	YLYRLFRKSNLKPFE	
Spike (S1 RBD)	511-525	VVLSFELLHAPATVC	
Spike (S1 RBD)	526-540	GPKKSTNLVKNKCVN	
Spike (S2)	721-735	SVTTEILPVSMTKTS	
Spike (S2)	746-760	STECSNLLLQYGSFC	
Spike (S2)	751-765	NLLLQYGSFCTQLNR	NLLLQYGSFCTQLKR
Spike (S2)	801-815	NFSQILPDPSKPSKR	
Spike (S2)	866-880	TDEMIAQYTSALLAG	
Spike (S2)	1171-1185	GINASVVNIQKEIDR	
Spike (S2)	1197-1210	LIDLQELGKYEQYI	
Spike (S2)	1206-1220	YEQYIKWPWYIWLGF	

Table S5B. B.1.1.529 (Omicron) peptide pool and the matched sequence peptide pool for Wuhan Hu-1.

	Position/mutation	Amino acid sequence
**Wuhan Hu-1 peptide pool (B.1.1.529)**		
Spike (S1)	A67V/del 69-70	PFFSNVTWFHAIHVSGTNGTK
Spike (S1)	G142D/del143-5	EFQFCNDPFLGVYYHKNNKSW
Spike (S1)	L212I	KIYSKHTPINLVRDLPQGFS
Spike (S1 RBD)	G339D	FPNITNLCPFGEVFNATRFA
Spike (S1 RBD)	S371L/S373P/S375F	VADYSVLYNSASFSTFKCYGV
Spike (S1 RBD)	K417N	RQIAPGQTGKIADYNYKLPD
Spike (S1 RBD)	N440K/G446S	CVIAWNSNNLDSKVGGNYNY
Spike (S1 RBD)	S477N/T478K/E484A	EIYQAGSTPCNGVEGFNCYF
Spike (S1 RBD)	Q493R/G496S/Q498R/N501Y/Y505H	FPLQSYGFQPTNGVGYQPYR
Spike (S1 RBD)	T547K	CVNFNFNGLTGTGVLTESNK
Spike (S1)	D614G	SNQVAVLYQDVNCTEVPVAI
Spike (S1)	H655Y	RAGCLIGAEHVNNSYECDIP
Spike (S1/S2)	N679K/P681H	CASYQTQTNSPRRARSVASQ
Spike (S2)	N764K	QYGSFCTQLNRALTGIAVEQ
Spike (S2)	D796Y	KQIYKTPPIKDFGGFNFSQI
Spike (S2)	N856K	RDLICAQKFNGLTVLPPLLT
Spike (S2)	Q954H	LGKLQDVVNQNAQALNTLV
Spike (S2)	N969K/L981F	KQLSSNFGAISSVLNDILSR
**B.1.1.529 (Omicron) variant peptide pool**		
Spike (S1)	A67V/HV69-70 del	PFFSNVTWFHVISGTNGTKRF
Spike (S1)	G142D/del143-5	EFQFCNDPFLDHKNNKSWMES
Spike (S1)	del211/L212I/ins214EPE	KIYSKHTPIIVREPEDLPQG
Spike (S1 RBD)	G339D	FPNITNLCPFDEVFNATRFA
Spike (S1 RBD)	S371L/S373P/S375F	VADYSVLYNLAPFFTFKCYGV
Spike (S1 RBD)	K417N	RQIAPGQTGNIADYNYKLPD
Spike (S1 RBD)	N440K/G446S	CVIAWNSNKLDSKVSGNYNY
Spike (S1 RBD)	S477N/T478K/E484A	EIYQAGNKPCNGVAGFNCYF
Spike (S1 RBD)	Q493R/G496S/Q498R/N501Y/Y505H	FPLRSYSFRPTYGVGHQPYR
Spike (S1 RBD)	T547K	CVNFNFNGLKGTGVLTESNK
Spike (S1)	D614G	SNQVAVLYQGVNCTEVPVAI
Spike (S1)	H655Y	RAGCLIGAEYVNNSYECDIP
Spike (S1/S2)	N679K/P681H	CASYQTQTKSHRRARSVASQ
Spike (S2)	N764K	QYGSFCTQLKRALTGIAVEQ
Spike (S2)	D796Y	KQIYKTPPIKYFGGFNFSQI
Spike (S2)	N856K	RDLICAQKFKGLTVLPPLLT
Spike (S2)	Q954H	LGKLQDVVNHNAQALNTLV
Spike (S2)	N969K/L981F	KQLSSKFGAISSVLNDIFSR

Table S6. HCW recruited at a median of 14 weeks after the third BNT162b2 vaccine dose with laboratory confirmed SARS-CoV-2 infection during the first Wuhan Hu-1 and fourth B.1.1.529 (Omicron) UK waves and infection naïve HCW.

	Total	SARS-CoV-2 infection naïve<br><br>SARS-CoV-2 PCR negative<br><br>Roche S1-RBD Ab and N Ab negative	SARS-CoV-2 infection during the first UK Wuhan Hu-1 wave	SARS-CoV-2 infected during the fourth UK B.1.1.529 (Omicron) wave	SARS-CoV-2 infected during the first UK Wuhan Hu-1 and fourth B.1.1.529 (Omicron) UK wave
HCW<br><br>n (% of total)	32 (100)	11 (34)	4 (13)	11 (34)	6 (19)
Mean age (range)	41<br>(23-61)	49<br>(31-61)	44<br>(37-48)	32<br>(23-57)	41<br>(31-59)
Gender:					
Female n (%)	20 (63)	8 (73)	2 (50)	8 (73)	2 (33)
Ethnicity:					
White n (%)	26 (81)	9 (82)	3 (75)	8 (73)	6 (100)
Minority ethnic group (UK) n (%)	6 (19)	2 (18)	1 (25)	3 (27)	0 (0)
BNT162b2 COVID-19 vaccine, n (%)	31 (97)	10 (91)	4 (100)	11 (100)	6 (100)
Number of days since third vaccine dose Median (IQR)	101 (25)	115 (30)	117 (16)	91 (45)	96 (23)

Abbreviations: Ab, antibody; HCW, healthcare worker; IQR, inter-quartile range; N, nucleocapsid; RBD, receptor binding domain; S1, subunit 1

Table S7. HCW recruited at a median of 14 weeks after the third BNT162b2 vaccine dose during the B.1.1.529 (Omicron) wave.

Abbreviations: F, Female; HCW, health care worker; M, Male; N, nucleocapsid; S1, Spike subunit 1; RBD, receptor binding domain.

Date recruited	Age	Sex	Date of 3rd BNT162B2 vaccine dose	Date PCR positive	N Ab (COI)	N Ab (COI) (prior)	Date of N Ab	S1 RBD Ab (U/ml)	S1 RBD Ab (prior)	Date of S1 RBD	S1 RBD (B.1.1.529) Ab (AU/ml)	Symptoms reported	Infection status during the B.1.1.52 (Omicron) wave	SARS-CoV-2 previous infection status	Date of previous SARS-CoV-2 infection Month/Y
28.1.22	56	F	4.10.21	-	0	0	19.10.21	14000	47263	19.10.21	16759	-	Uninfected	Infection naïve	-
28.1.22	49	M	23.9.21	-	0	0	18.10.21	5143	24750	18.10.21	8357	-	Uninfected	Infection naïve	-
21.1.22	60	F	28.9.21	-	0	0	19.10.21	3791	10270	19.10.21	8430	-	Uninfected	Infection naïve	-
28.1.22	46	F	11.10.21	-	0	0	2.11.21	17443	61535	2.11.21	20484	-	Uninfected	Infection naïve	-
28.1.22	51	F	21.9.21	-	0	0	8.4.21	11892	20298	8.4.21	15962	-	Uninfected	Infection naïve	-
21.1.22	32	F	20.10.21	-	0	0	5.11.21	8429	29566	5.11.21	11831	-	Uninfected	Infection naïve	-
21.1.22	44	F	13.10.21	-	0	0	25.10.21	4440	25037	25.10.21	4662	-	Uninfected	Infection naïve	-
21.1.22	31	M	15.10.21	-	0	0	25.10.21	19664	47428	25.10.21	17851	-	Uninfected	Infection naïve	-
31.1.22	57	F	22.9.21	-	0	0	26.10.21	4432	10603	26.10.21	7640	-	Uninfected	Infection naïve	-
31.1.22	55	F	25.9.21	-	0	0	26.10.21	6067	30354	26.10.21	14839	-	Uninfected	Infection naïve	-
31.1.22	61	M	10.11.21	-	0	0	26.10.21	9600	771	26.10.21	16151	-	Uninfected	Infection naïve	-
28.1.22	48	F	11.10.21	-	19.4	21.0	25.10.21	4411	13974	25.10.21	6619	-	Uninfected	Wuhan Hu-1	03/20
28.1.22	37	F	7.10.21	-	13.5	15.5	29.10.21	24055	47112	29.10.21	34556	-	Uninfected	Wuhan Hu-1	04/20
28.1.22	48	M	22.9.21	-	0	0	18.10.21	7917	19807	18.10.21	11169	-	Uninfected	Wuhan Hu-1	03/20
28.1.22	46	M	29.9.21	-	2.0	1.4	19.10.21	2383	6181	19.10.21	3412	-	Uninfected	Wuhan Hu-1	03/20
7.1.22	26	F	13.10.21	27.12.21	1.1	0	9.4.21	20391	16527	9.4.21	27069	Cough/sore throat	Infected	Infection naïve	-
6.1.22	57	F	27.9.21	29.12.21	0	0	14.4.21	26170	18832	14.4.21	32304	Cough<br>Runny nose	Infected	Infection naïve	-
7.1.22	25	F	29.9.21	20.12.21	0	0	15.4.21	24515	14337	15.4.21	27172	Fatigue<br>Myalgia<br>Runny nose<br>Sore throat	Infected	Infection naïve	-
12.1.22	23	F	13.10.21	29.12.21	27.4	0	15.4.21	34464	74877	15.4.21	47967	Loss of smell<br>Fatigue<br>Myalgia<br>Runny nose<br>Sore throat	Infected	Infection naïve	-
10.1.22	35	M	16.11.21	22.12.21	2.76	0	12.4.21	41060	9928	12.4.21	43286	Fever<br>Shortness of breath<br>Fatigue<br>Myalgia<br>Sore throat	Infected	Infection naïve	-
10.1.22	29	F	11.12.21	19.12.21	2.42	0	18.10.21	42103	2299	18.10.21	32509	Cough<br>Runny nose<br>Sore throat	Infected	Infection naïve	-
21.1.22	29	M	8.10.21	18.12.21	4.10	0	7.4.21	33392	15364	7.4.21	38440	Cough<br>Fever<br>Fatigue<br>Myalgia<br>Sore throat	Infected	Infection naïve	-
21.1.22	23	F	13.10.21	8.1.22	50.9	0	14.4.21	33569	20024	14.4.21	17775	Cough	Infected	Infection naïve	-
6.1.22	35	F	4.10.21	21.12.21	6.37	0	7.4.21	51337	46581	7.4.21	105695	Shortness of breath<br>Fatigue<br>Myalgia	Infected	Infection naïve	-
12.1.22	40	F	12.12.21	22.12.21	1.66	0	15.4.21	23228	6359	15.4.21	40102	Asymptomatic	Infected	Infection naïve	-
10.1.22	35	M	13.10.21	29.12.21	2.33	0	20.4.21	34250	12441	20.4.21	33181	Fever<br>Loss of smell<br>shortness of breath<br>Fatigue<br>GI symptoms<br>Myalgia	Infected	Infection naïve	-
7.1.22	50	M	8.10.21	13.12.21	187	2.76	22.10.21	10660	22200	22.10.21	6498	Cough<br>Fatigue<br>GI symptoms	Infected	Wuhan Hu-1	03/20
21.1.22	35	M	10.10.21	-	37.9	0	25.10.21	7152	10651	25.10.21	3382	Asymptomatic	Infected	Wuhan Hu-1	03/20
7.1.22	35	F	19.10.21	20.12.21	234	0	29.10.21	14070	28936	29.10.21	12128	Asymptomatic	Infected	Wuhan Hu-1	03/20
6.1.22	59	M	25.9.21	19.12.21	152	7.32	29.10.21	14496	9555	29.10.21	16449	Cough<br>Sore throat	Infected	Wuhan Hu-1	03/20
6.1.22	38	M	20.10.21	21.12.21	148	1.49	16.11.21	7772	8885	16.11.21	13438	Cough	Infected	Wuhan Hu-1	03/20
28.1.22	31	F	19.10.21	15.1.22	104	4.69	16.4.21	4408	9215	16.4.21	5177	Cough<br>Fatigue<br>GI symptoms<br>Myalgia	Infected	Wuhan Hu-1	04/20

Table S8. Spike mutations in VOC viral isolates used in this study. Mutations in the receptor binding domain (RBD) have been highlighted in grey. Mutations in the S2 region are underlined.

**Lineage**	**Spike Mutations (Mutations in RBD)**
Wuhan Hu-1	S247R
B.1.1.7	H69del, V70del, Y145del, H146H/R, N501Y, A570D, D614G, P681H, T716I, S982A, D1118H
B.1.351	D80A, D215G, K417N, E484K, N501Y, D614G, A701V, nucleotides 22281-22289
P.1	L18F, T20N, P26S, D138Y, R190S, K417T, E484K, N501Y, D614G, H655Y, T1027I, V1176F
B.1.617.2	T19R, L452R, T478K, D614G, P681R, D950N, nucleotides 22029-22034
B.1.1.529	A67V, H69del, V70del, T95I, G142D, V143del, Y144del, Y145del, N211I, L212I, G339D, S371L, S373P, S375F, K417N, N440K, G446S, S477N, T478K, E484A, Q493R, G496S, Q498R, N501Y, Y505H, T547K, D614G, H655Y, N679K, P681H, N764K, D796Y, N856K, Q954H, N969K, L981F

Table S9. *In silico* predictions of B.1.1.529 (Omicron) variant peptide binding to common UK HLAII alleles. NetMHCIIpan epitope predictions for binding of B.1.1.529 (Omicron) variant mutated regions to common UK HLAII alleles. Weak binding is annotated blue, strong binding is annotated yellow and the HLA binding core in each instance is shown with the mutated amino acid(s) marked in red

	Mutation	Sequence	NetMHCIIpan predicted HLA binding core with B.1.1.529 mutation indicated						
			DRB1*0101	DRB1*0301	DRB1*0401	DRB1*0701	DRB1*1101	DRB1*1301	DRB1*1501
Spike	A67V/Δ69-70	Wuhan Hu-1	VTWFH**A**I**HV**	-	I**HV**SGTNGT	VTWFH**A**I**HV**	WFH**A**I**HV**SG	VTWFH**A**I**HV**	VTWFH**A**I**HV**
		B.1.1.529	FH**V**ISGTNG	-	FH**V**ISGTNG	FH**V**ISGTNG	FH**V**ISGTNG	-	VTWFH**V**ISG
	T95I	Wuhan Hu-1	VYFAS**T**EKS	-	VYFAS**T**EKS	YFAS**T**EKSN	FAS**T**EKSNI	-	-
		B.1.1.529	-	-	VYFAS**I**EKS	YFAS**I**EKSN	FAS**I**EKSNI	-	-
	G142D/del143-145	Wuhan Hu-1	-	-	**VYY**HKNNKS	-	**Y**HKNNKSWM	**VYY**HKNNKS	**VYY**HKNNKS
		B.1.1.529	-	-	FL**D**HKNNKS	-	FL**D**HKNNKS	-	FL**D**HKNNKS
	del211/L212I/ins214EPE	Wuhan Hu-1	YSKHTPI**NL**	**L**V**RD**LPQGF	**INL**V**RD**LPQ	YSKHTPI**NL**	**INL**V**RD**LPQ	**INL**V**RD**LPQ	**INL**V**RD**LPQ
		B.1.1.529	YSKHTPI**I**V	-	-	YSKHTPI**I**V	-	IYSKHTPI**I**	IYSKHTPI**I**
	G339D	Wuhan Hu-1	-	-	-	-	-	-	-
		B.1.1.529	-	-	-	-	-	-	-
	S371L/S373P/S375F	Wuhan Hu-1	YSVLYN**S**A**S**	-	YSVLYN**S**A**S**	LYN**S**A**S**F**S**T	YSVLYN**S**A**S**	-	F**S**TFKCYGV
		B.1.1.529	F**F**TFKCYGV	-	-	F**F**TFKCYGV	-	-	**L**A**P**F**F**TFKC
	K417N	Wuhan Hu-1	-	-	-	-	-	-	-
		B.1.1.529	-	-	-	-	-	-	-
	N440K/**G446S**	Wuhan Hu-1	VIAWNSN**N**L	IAWNSN**N**LD	IAWNSN**N**LD	VIAWNSN**N**L	-	VIAWNSN**N**L	VIAWNSN**N**L
		B.1.1.529	VIAWNSN**K**L	-	VIAWNSN**K**L	VIAWNSN**K**L	-	VIAWNSN**K**L	VIAWNSN**K**L
	S477N/T478K/E484A	Wuhan Hu-1	IYQAG**ST**PC	-	IYQAG**ST**PC	IYQAG**ST**PC	-	-	IYQAG**ST**PC
		B.1.1.529	IYQAG**NK**PC	-	IYQAG**NK**PC	-	-	-	IYQAG**NK**PC
	Q493R/G496S/Q498R/N501Y/Y505H	Wuhan Hu-1	Y**G**F**Q**PT**N**GV	-	Y**G**F**Q**PT**N**GV	F**Q**PT**N**GVG**Y**	-	-	**Y**QPYRVVVL
		B.1.1.529	YFPL**R**SY**S**F	-	-	F**R**PT**Y**GVG**H**	-	-	Y**S**F**R**PT**Y**GV
	T547K	Wuhan Hu-1	FNGL**T**GTGV	-	-	FNGL**T**GTGV	-	-	-
		B.1.1.529	FNGL**K**GTGV	-	-	FNGL**K**GTGV	-	-	-
	D614G	Wuhan Hu-1	-	-	-	YQ**D**VNCTEV	-	-	AVLYQ**D**VNC
		B.1.1.529	-	-	-	YQ**G**VNCTEV	-	-	AVLYQ**G**VNC
	H655Y	Wuhan Hu-1	-	-	IGAE**H**VNNS	-	-	-	-
		B.1.1.529	-	-	-	-	-	-	-
	N679K/P681H	Wuhan Hu-1	-	-	YQTQT**N**S**P**R	-	-	-	-
		B.1.1.529	-	YQTQT**K**S**H**R	-	-	YQTQT**K**S**H**R	YQTQT**K**S**H**R	-
	N764K	Wuhan Hu-1	**N**RALTGIAV	-	-	-	-	-	-
		B.1.1.529	**K**RALTGIAV	-	-	-	-	-	-
	D796Y	Wuhan Hu-1	-	-	IYKTPPIK**D**	IYKTPPIK**D**	-	IK**D**FGGFNF	IK**D**FGGFNF
		B.1.1.529	IK**Y**FGGFNF	IYKTPPIK**Y**	-	IYKTPPIK**Y**	-	IYKTPPIK**Y**	IK**Y**FGGFNF
	N856K	Wuhan Hu-1	-	-	-	-	LICAQKF**N**G	-	-
		B.1.1.529	F**K**GLTVLPP	-	F**K**GLTVLPP	-	F**K**GLTVLPP	LICAQKF**K**G	-
	Q954H	Wuhan Hu-1	VVN**Q**NAQAL	-	LQDVVN**Q**NA	-	-	-	VVN**Q**NAQAL
		B.1.1.529	-	-	LQDVVN**H**NA	VVN**H**NAQAL	-	VVN**H**NAQAL	VVN**H**NAQAL
	N969K	Wuhan Hu-1	VKQLSS**N**FG	-	VKQLSS**N**FG	-	-	-	VKQLSS**N**FG
		B.1.1.529	VKQLSS**K**FG	-	-	-	-	-	VKQLSS**K**FG
	L981F	Wuhan Hu-1	-	VLNDI**L**SRL	-	-	**L**SRLDKVEA	**L**SRLDKVEA	ISSVLNDI**L**
		B.1.1.529	**F**SRLDKVEA	-	**F**SRLDKVEA	-	**F**SRLDKVEA	**F**SRLDKVEA	-

Table S10. *In silico* predictions of B.1.1.529 (Omicron) variant peptide binding to common UK HLA-A alleles. NetMHCpan epitope predictions for binding of B.1.1.529 (Omicron) variant mutated regions to common UK HLA alleles. Weak binding is annotated blue, strong binding is annotated yellow and the HLA binding core in each instance is shown with the mutated amino acid(s) marked in red.

			NetMHCpan predicted HLA binding core with B.1.1.529 mutation indicated				
	Mutation	Sequence	A01:01	A02:01	A03:01	A11:01	A24:02
Spike	A67V/Δ69-70	Wuhan Hu-1	-	VTWFHAIHV	HVSGTNGTK	HVSGTNGTK	-
		B.1.1.529	-	-	HVISGTNGTK	HVISGTNGTK	-
	T95I	Wuhan Hu-1	STEKSNIIR	-	GVYFASTEK	GVYFASTEK	-
		B.1.1.529	-	-	GVYFASIEK	GVYFASIEK	-
	G142D/del143-145	Wuhan Hu-1	FCNDPFLGVY	FCNDPFLGV	GVYYHKNNK	GVYYHKNNK	YYHKNNKSW
		B.1.1.529	FLDHKNNKSW	-	-	-	-
	del211/L212I/ins214EPE	Wuhan Hu-1	-	-	-	-	IYSKHTPINL
		B.1.1.529	-	-	-	-	IYSKHTPII
	G339D	Wuhan Hu-1	-	NLCPFGEVFNA	-	-	PFGEVFNATRF
		B.1.1.529	-	NLCPFDEVFNA	-	-	PFDEVFNATRF
	S371L/S373P/S375F	Wuhan Hu-1	NSASFSTFKCY	-	NSASFSTFK	NSASFSTFK	LYNSASFSTF
		B.1.1.529	NLAPFFTFKCY	VLYNLAPFF	NLAPFFTFK	NLAPFFTFK	LYNLAPFFTF
	K417N	Wuhan Hu-1	QTGKIADYNY	KIADYNYKL	RQIAPGQTGK	RQIAPGQTGK	KIADYNYKL
		B.1.1.529	QTGNIADYNY	NIADYNYKL	-	QTGNIADYNYK	-
	N440K/G446S	Wuhan Hu-1	NLDSKVGGNY	-	-	-	GGNYNYLYRLF
		B.1.1.529	VSGNYNYLY	KVSGNYNYL	CVIAWNSNK	CVIAWNSNK	SGNYNYLYRLF
	S477N/T478K/E484A	Wuhan Hu-1	-	YQAGSTPCNGV	-	-	-
		B.1.1.529	-	-	STEIYQAGNK	STEIYQAGNK	-
	Q493R/G496S/Q498R/N501Y/Y505H	Wuhan Hu-1	PTNGVGYQPY	YQPYRVVVL	-	-	YFPLQSYGF
		B.1.1.529	PTYGVGHQPY	GVGHQPYRV	RSYSFRPTY	RSYSFRPTY	YFPLRSYGF
	T547K	Wuhan Hu-1	-	-	-	-	-
		B.1.1.529	-	-	CVNFNFNGLK	CVNFNFNGLK	-
	D614G	Wuhan Hu-1	-	VLYQDVNCTEV	-	-	-
		B.1.1.529	-	VLYQGVNCTEV	-	-	-
	H655Y	Wuhan Hu-1	GAEHVNNSY	-	-	-	-
		B.1.1.529	GAEYVNNSY	-	-	-	-
	N679K/P681H	Wuhan Hu-1	-	-	QTNSPRRAR	ASYQTQTNSPR	-
		B.1.1.529	-	-	ASYQTQTK	ASYQTQTKSHR	-
	N764K	Wuhan Hu-1	-	QLNRALTGI	GSFCTQLNR	GSFCTQLNR	-
		B.1.1.529	-	-	LQYGSFCTQLK	GSFCTQLKR	-
	D796Y	Wuhan Hu-1	-	-	-	-	IYKTPPIKDF
		B.1.1.529	QIYKTPPIKY	-	QIYKTPPIKY	QIYKTPPIKY	IYKTPPIKYF
	N856K	Wuhan Hu-1	-	-	-	-	-
		B.1.1.529	-	-	RDLICAQKFK	-	-
	Q954H	Wuhan Hu-1	-	KLQDVVNQNA	-	-	-
		B.1.1.529	-	KLQDVVNHNA	-	-	-
	N969K	Wuhan Hu-1	-	-	-	-	NFGAISSVL
		B.1.1.529	-	-	LNTLVKQLSSK	TLVKQLSSK	KFGAISSVL
	L981F	Wuhan Hu-1	-	VLNDILSRL	VLNDILSRLDK	SVLNDILSR	-
		B.1.1.529	-	VLNDIFSRL	VLNDIFSRLDK	SVLNDIFSR	VLNDIFSRL

Table S11. ***In silico*** **predictions of B.1.1.529 (Omicron) variant peptide binding to common UK HLA-B alleles**. NetMHCpan epitope predictions for binding of B.1.1.529 (Omicron) variant mutated regions to common UK HLA alleles. Weak binding is annotated blue, strong binding is annotated yellow and the HLA binding core in each instance is shown with the mutated amino acid(s) marked in red.

			NetMHCpan predicted HLA binding core with B.1.1.529 mutation indicated						
	Mutation	Sequence	B07:02	B08:01	B15:01	B35:01	B40:01	B44:02	B44:03
	A67V/Δ69-70	Wuhan Hu-1	-	-	-	-	-	-	-
		B.1.1.529	-	-	-	-	-	-	-
	T95I	Wuhan Hu-1	-	-	-	-	-	TEKSNIIRGW	TEKSNIIRGW
		B.1.1.529	-	-	-	-	-	IEKSNIIRGW	IEKSNIIRGW
	G142D/del143-145	Wuhan Hu-1	-	-	FCNDPFLGVY	DPFLGVYY	-	-	-
		B.1.1.529	-	-	-	-	-	LDHKNNKSW	LDHKNNKSW
	del211/L212I/ins214EPE	Wuhan Hu-1	TPINLVRDL	YSKHTPINL	LVRDLPQGF	TPINLVRDL	RDLPQGFSAL	-	-
		B.1.1.529	REPEDLPQGF	-	-	EPEDLPQGF	REPEDLPQGF	REPEDLPQGF	REPEDLPQGF
	G339D	Wuhan Hu-1	-	-	NLCPFGEVF	NLCPFGEVF	GEVFNATRF	GEVFNATRF	GEVFNATRF
		B.1.1.529	-	-	NLCPFDEVF	NLCPFDEVF	DEVFNATRF	DEVFNATRF	DEVFNATRF
	S371L/S373P/S375F	Wuhan Hu-1	-	-	ASFSTFKCY	SVLYNSASF	-	-	ASFSTFKCY
		B.1.1.529	APFFTFKCY	-	VLYNLAPFF	APFFTFKCY	-	-	APFFTFKCY
	K417N	Wuhan Hu-1	APGQTGKIA	-	GQTGKIADY	-	RQIAPGQTGKI	-	-
		B.1.1.529	APGQTGNIA	-	GQTGNIADY	APGQTGNIADY	-	-	-
	N440K/G446S	Wuhan Hu-1	-	-	SKVGGNYNY	-	-	-	LDSKVGGNY
		B.1.1.529	-	-	KLDSKVSGNY	-	-	-	SKVSGNYNY
	S477N/T478K/E484A	Wuhan Hu-1	TPCNGVEGF	-	-	TPCNGVEGF	-	-	-
		B.1.1.529	KPCNGVAGF	-	GVAGFNCYF	KPCNGVAGF	-	-	-
	Q493R/G496S/Q498R/N501Y/Y505H	Wuhan Hu-1	-	YQPYRVVVL	FQPTNGVGY	NCYFPLQSY	YQPYRVVVL	-	-
Spike		B.1.1.529	RPTYGVGHQP	HQPYRVVVL	RSYSFRPTY	FPLRSYS	HQPYRVVVL	-	-
	T547K	Wuhan Hu-1	-	-	-	-	-	-	-
		B.1.1.529	-	-	-	-	-	-	-
	D614G	Wuhan Hu-1	-	-	-	-	-	-	-
		B.1.1.529	-	-	-	-	-	-	-
	H655Y	Wuhan Hu-1	-	-	IGAEHVNNSY	GAEHVNNSY	AEHVNNSY	AEHVNNSY	AEHVNNSY
		B.1.1.529	-	-	GAEYVNNSY	GAEYVNNSY	AEYVNNSY	AEYVNNSY	AEYVNNSY
	N679K/P681H	Wuhan Hu-1	SPRRARSVA	SPRRARSV	-	-	-	-	-
		B.1.1.529	-	KSHRRARSV	-	-	-	-	-
	N764K	Wuhan Hu-1	-	-	-	-	-	-	-
		B.1.1.529	-	FCTQLKRAL	-	-	-	-	-
	D796Y	Wuhan Hu-1	-	-	-	TPPIKDFGGF	-	-	-
		B.1.1.529	-	-	KQIYKTPPIKY	-	-	KQIYKTPPIKY	KQIYKTPPIKY
	N856K	Wuhan Hu-1	-	ICAQKFNGL	AQKFNGLTVL	-	AQKFNGLTVL	-	-
		B.1.1.529	-	AQKFKGLTV	AQKFKGLTVL	-	AQKFKGLTVL	-	-
	Q954H	Wuhan Hu-1	VVNQNAQAL	VVNQNAQAL	VVNQNAQAL	VVNQNAQAL	-	-	-
		B.1.1.529	VVNHNAQAL	VVNHNAQAL	-	-	-	-	-
	N969K	Wuhan Hu-1	-	LVKQLSSNF	LVKQLSSNF	LVKQLSSNF	-	-	-
		B.1.1.529	-	QLSSKFGAI	LVKQLSSKF	LVKQLSSKF	-	-	-
	L981F	Wuhan Hu-1	-	DILSRLDKV	VLNDILSRL	-	-	-	-
		B.1.1.529	-	DIFSRLDKV	VLNDIFSRL	-	-	-	-

Table S12. ***In silico*** **predictions of B.1.1.529 (Omicron) variant peptide binding to common UK HLA-C alleles**. NetMHCpan epitope predictions for binding of B.1.1.529 (Omicron) variant mutated regions to common UK HLA alleles. Weak binding is annotated blue, strong binding is annotated yellow and the HLA binding core in each instance is shown with the mutated amino acid(s) marked in red.

			NetMHCpan predicted HLA binding core with B.1.1.529 mutation indicated						
	Mutation	Sequence	C03:03	C03:04	C04:01	C05:01	C06:02	C07:01	C07:02
Spike	A67V/Δ69-70	Wuhan Hu-1	-	-	-	-	VTWFHAIHV	-	-
		B.1.1.529	-	-	-	-	-	-	-
	T95I	Wuhan Hu-1	FASTEKSNI	FASTEKSNI	-	FASTEKSNI	FASTEKSNI	-	-
		B.1.1.529	FASIEKSNI	FASIEKSNI	-	-	ASIEKSNII	-	-
	G142D/del143-145	Wuhan Hu-1	FCNDPFLGV	FCNDPFLGV	FCNDPFLGV	FCNDPFLGV	YYHKNNKSW	YYHKNNKSW	YYHKNNKSW
		B.1.1.529	-	-	-	FLDHKNNKSW	-	-	-
	del211/L212I/ins214EPE	Wuhan Hu-1	YSKHTPINL	YSKHTPINL	YSKHTPINL	YSKHTPINL	SKHTPINLV	YSKHTPINL	YSKHTPINL
		B.1.1.529	YSKHTPIIV	YSKHTPIIV	IYSKHTPII	EPEDLPQGF	YSKHTPIIV	IYSKHTPII	IYSKHTPII
	G339D	Wuhan Hu-1	-	-	-	-	-	-	-
		B.1.1.529	-	-	PFDEVFNATRF	-	-	-	-
	S371L/S373P/S375F	Wuhan Hu-1	SVLYNSASF	SVLYNSASF	YNSASFSTF	-	YNSASFSTF	YNSASFSTF	YNSASFSTF
		B.1.1.529	YNLAPFFTF	YNLAPFFTF	-	-	-	-	LYNLAPFF
	K417N	Wuhan Hu-1	KIADYNYKL	KIADYNYKL	KIADYNYKL	KIADYNYKL	KIADYNYKL	KIADYNYKL	KIADYNYKL
		B.1.1.529	NIADYNYKL	NIADYNYKL	NIADYNYKL	NIADYNYKL	NIADYNYKL	NIADYNYKL	NIADYNYKL
	N440K/G446S	Wuhan Hu-1	IAWNSNNL	IAWNSNNL	-	-	-	SKVGGNYNY	SKVGGNYNY
		B.1.1.529	IAWNSNKL	IAWNSNKL	KVSGNYNYL	KVSGNYNYL	KVSGNYNYL	SKVSGNYNY	SKVSGNYNY
	S477N/T478K/E484A	Wuhan Hu-1	-	-	-	GVEGFNCYF	-	-	-
		B.1.1.529	-	-	-	-	-	-	-
	Q493R/G496S/Q498R/N501Y/Y505H	Wuhan Hu-1	YQPYRVVVL	YQPYRVVVL	YFPLQSYGF	YQPYRVVVL	YQPYRVVVL	YQPYRVVVL	YQPYRVVVL
		B.1.1.529	HQPYRVVVL	HQPYRVVVL	YFPLRSYSF	HQPYRVVVL	HQPYRVVVL	RSYSFRPTY	YFPLRSYSF
	T547K	Wuhan Hu-1	-	-	-	-	-	-	-
		B.1.1.529	-	-	-	-	-	-	-
	D614G	Wuhan Hu-1	-	-	YQDVNCTEV	YQDVNCTEV	-	-	-
		B.1.1.529	-	-	-	-	-	-	-
	H655Y	Wuhan Hu-1	-	-	-	-	-	-	-
		B.1.1.529	-	-	-	-	-	TRAGCLIGAEY	TRAGCLIGAEY
	N679K/P681H	Wuhan Hu-1	-	-	-	-	NSPRRARSV	-	-
		B.1.1.529	-	-	-	-	KSHRRARSV	-	SYQTQTKSH
	N764K	Wuhan Hu-1	FCTQLNRAL	FCTQLNRAL	-	-	NRALTGIAV	NRALTGIAV	NRALTGIAV
		B.1.1.529	FCTQLKRAL	FCTQLKRAL	-	-	KRALTGIAV	KRALTGIAV	KRALTGIAV
	D796Y	Wuhan Hu-1	-	-	IKDFGGFNF	IKDFGGFNF	YKTPPIKDF	YKTPPIKDF	YKTPPIKDF
		B.1.1.529	YKTPPIKYF	YKTPPIKYF	IYKTPPIKY	-	YKTPPIKYF	YKTPPIKYF	IYKTPPIKY
	N856K	Wuhan Hu-1	-	-	KFNGLTVL	-	QKFNGLTVL	QKFNGLTVL	QKFNGLTVL
		B.1.1.529	-	-	-	-	QKFKGLTVL	QKFKGLTVL	QKFKGLTVL
	Q954H	Wuhan Hu-1	VVNQNAQAL	VVNQNAQAL	VVNQNAQAL	VVNQNAQAL	VVNQNAQAL	-	-
		B.1.1.529	VVNHNAQAL	VVNHNAQAL	VVNHNAQAL	VVNHNAQAL	VVNHNAQAL	-	-
	N969K	Wuhan Hu-1	-	-	NFGAISSVL	-	SNFGAISSV	SNFGAISSV	-
		B.1.1.529	-	-	KFGAISSVL	-	SKFGAISSV	SKFGAISSV	SKFGAISSV
	L981F	Wuhan Hu-1	VLNDILSRL	VLNDILSRL	VLNDILSRL	VLNDILSRL	VLNDILSRL	VLNDILSRL	VLNDILSRL
		B.1.1.529	VLNDIFSRL	VLNDIFSRL	VLNDIFSRL	VLNDIFSRL	VLNDIFSRL	VLNDIFSRL	VLNDIFSRL

Table S13. *In silico* predictions of BA.2 variant peptide binding to common UK HLAII alleles. NetMHCIIpan epitope predictions for binding of BA.2 variant mutated regions to common UKII HLA alleles. Weak binding is annotated blue, strong binding is annotated yellow and the HLA binding core in each instance is shown with the mutated amino acid(s) marked in red.

			NetMHCIIpan predicted HLA binding core with BA.2 mutation indicated						
	Mutation	Sequence	DRB1*0101	DRB1*0301	DRB1*0401	DRB1*0701	DRB1*1101	DRB1*1301	DRB1*1501
Spike	T19I/24-26del/A27S	Wuhan Hu-1	-	-	-	-	-	-	-
		BA.2	-	-	-	-	VNLITRTQS	-	-
	G142D	Wuhan Hu-1	-	-	-	-	-	-	-
		BA.2	-	-	-	-	-	-	-
	V213G	Wuhan Hu-1	-	LVRDLPQGF	INLVRDLPQ	-	-	-	INLVRDLPQ
		BA.2	-	LGRDLPQGF	-	-	-	-	-
	G339D	Wuhan Hu-1	-	-	-	-	-	-	-
		BA.2	-	-	-	-	-	-	-
	S371F/S373P/S375F/T376A	Wuhan Hu-1	YSVLYNSAS	-	YSVLYNSAS	FSTFKCYGV	-	-	FSTFKCYGV
		BA.2	-	-	-	FFAFKCYGV	-	-	FFAFKCYGV
	D405N/R408S	Wuhan Hu-1	-	IRGDEVRQI	-	-	-	FVIRGDEVR	-
		BA.2	-	-	FVIRGNEVS	-	FVIRGNEVS	-	-
	K417N	Wuhan Hu-1	-	-	-	-	-	-	-
		BA.2	-	-	-	-	-	-	-
	N440K	Wuhan Hu-1	-	-	IAWNSNNLD	-	-	VIAWNSNNL	VIAWNSNNL
		BA.2	-	-	-	VIAWNSNKL	-	VIAWNSNKL	VIAWNSNKL
	S477N/T478K/E484A	Wuhan Hu-1	IYQAGSTPC	-	IYQAGSTPC	IYQAGSTPC	-	-	IYQAGSTPC
		BA.2	IYQAGNKPC	-	IYQAGNKPC	-	-	-	IYQAGNKPC
	Q493R/Q498R/N501Y/Y505H	Wuhan Hu-1	YGFQPTNGV	-	YGFQPTNGV	FQPTNGVGY	-	-	YQPYRVVVL
		BA.2	-	-	-	FRPTYGVGH	-	-	-
	D614G	Wuhan Hu-1	-	-	-	YQDVNCTEV	-	-	AVLYQDVNC
		BA.2	-	-	-	YQGVNCTEV	-	-	AVLYQGVNC
	H655Y	Wuhan Hu-1	-	-	IGAEHVNNS	-	-	-	-
		BA.2	-	-	-	-	-	-	-
	N679K/P681H	Wuhan Hu-1	-	-	YQTQTNSPR	-	-	-	-
		BA.2	-	YQTQTKSHR	-	-	YQTQTKSHR	YQTQTKSHR	-
	N764K	Wuhan Hu-1	NRALTGIAV	-	-	-	-	-	-
		BA.2	KRALTGIAV	-	-	-	-	-	-
	D796Y	Wuhan Hu-1	-	-	IYKTPPIKD	IYKTPPIKD	-	IKDFGGFNF	IKDFGGFNF
		BA.2	IKYFGGFNF	IYKTPPIKY	-	IYKTPPIKY	-	IYKTPPIKY	IKYFGGFNF
	Q954H	Wuhan Hu-1	VVNQNAQAL	-	LQDVVNQNA	-	-	-	VVNQNAQAL
		BA.2	-	-	LQDVVNHNA	VVNHNAQAL	-	VVNHNAQAL	VVNHNAQAL
	N969K	Wuhan Hu-1	VKQLSSNFG	-	VKQLSSNFG	-	-	-	VKQLSSNFG
		BA.2	VKQLSSKFG	-	-	-	-	-	VKQLSSKFG

Table S14. ***In silico*** **predictions of BA.2 variant peptide binding to common UK HLA-A alleles.** NetMHCpan epitope predictions for binding of BA.2 variant mutated regions to common UK HLA alleles. Weak binding is annotated blue, strong binding is annotated yellow and the HLA binding core in each instance is shown with the mutated amino acid(s) marked in red.

			NetMHCpan predicted HLA binding core with BA.2 mutation indicated				
	**Mutation**	**Sequence**	**A01:01**	**A02:01**	**A03:01**	**A11:01**	**A24:02**
Spike	T19I/24-26del/A27S	Wuhan Hu-1	TTRTQLPPAY	-	-	SSQCVNLTTR	QLPPAYTNSF
		BA.2	NLITRTQSY	-	RTQSYTNSFTR	QSYTNSFTR	RTQSYTNSF
	G142D	Wuhan Hu-1	FCNDPFLGVY	FQFCNDPFLGV	GVYYHKNNK	GVYYHKNNK	GVYYHKNNKSW
		BA.2	FCNDPFLDVY	-	LDVYYHKNNK	DVYYHKNNK	DVYYHKNNKSW
	V213G	Wuhan Hu-1	-	-	-	-	IYSKHTPINLV
		BA.2	-	-	-	-	-
	G339D	Wuhan Hu-1	-	NLCPFGEVFNA	-	-	PFGEVFNATRF
		BA.2	-	NLCPFDEVFNA	-	-	PFDEVFNATRF
	S371F/S373P/S375F/T376A	Wuhan Hu-1	NSASFSTFKCY	-	NSASFSTFK	NSASFSTFK	LYNSASFSTF
		BA.2	-	VLYNFAPFFA	NFAPFFAFK	NFAPFFAFK	LYNFAPFFAF
	D405N/R408S	Wuhan Hu-1	-	-	RQIAPGQTGK	RQIAPGQTGK	-
		BA.2	-	-	-	-	-
	K417N	Wuhan Hu-1	QTGKIADYNY	KIADYNYKL	RQIAPGQTGK	RQIAPGQTGK	KIADYNYKL
		BA.2	QTGNIADYNY	NIADYNYKL	-	QTGNIADYNYK	-
	N440K	Wuhan Hu-1	NLDSKVGGNY	-	-	-	-
		BA.2	KLDSKVGGNY	-	CVIAWNSNK	CVIAWNSNK	-
	S477N/T478K/E484A	Wuhan Hu-1	-	YQAGSTPCNGV	-	-	-
		BA.2	-	-	STEIYQAGNK	STEIYQAGNK	-
	Q493R/Q498R/N501Y/Y505H	Wuhan Hu-1	PTNGVGYQPY	YQPYRVVVL	-	-	YFPLQSYGF
		BA.2	PTYGVGHQPY	GVGHQPYRV	RSYGFRPTY	RSYGFRPTY	YFPLRSYGF
	D614G	Wuhan Hu-1	-	VLYQDVNCTEV	-	-	-
		BA.2	-	VLYQGVNCTEV	-	-	-
	H655Y	Wuhan Hu-1	GAEHVNNSY	-	-	-	-
		BA.2	GAEYVNNSY	-	-	-	-
	N679K/P681H	Wuhan Hu-1	-	-	QTNSPRRAR	ASYQTQTNSPR	-
		BA.2	-	-	ASYQTQTK	ASYQTQTKSHR	-
	N764K	Wuhan Hu-1	-	QLNRALTGI	GSFCTQLNR	GSFCTQLNR	-
		BA.2	-	-	LQYGSFCTQLK	GSFCTQLKR	-
	D796Y	Wuhan Hu-1	-	-	-	-	IYKTPPIKDF
		BA.2	QIYKTPPIKY	-	QIYKTPPIKY	QIYKTPPIKY	IYKTPPIKYF
	Q954H	Wuhan Hu-1	-	KLQDVVNQNA	-	-	-
		BA.2	-	KLQDVVNHNA	-	-	-
	N969K	Wuhan Hu-1	-	-	-	-	NFGAISSVL
		BA.2	-	-	LNTLVKQLSSK	TLVKQLSSK	KFGAISSVL

Table S15. ***In silico*** **predictions of BA.2 variant peptide binding to common UK HLA-B alleles**. NetMHCpan epitope predictions for binding of BA.2 variant mutated regions to common UK HLA alleles. Weak binding is annotated blue, strong binding is annotated yellow and the HLA binding core in each instance is shown with the mutated amino acid(s) marked in red.

			NetMHCpan predicted HLA binding core with BA.2 mutation indicated						
	Mutation	Sequence	B07:02	B08:01	B15:01	B35:01	B40:01	B44:02	B44:03
	T19I/24-26del/A27S	Wuhan Hu-1	LPPAYTNSF	NLTTRTQL	TQLPPAYTNSF	LPPAYTNSF	-	-	TQLPPAYTNSF
		BA.2	RTQSYTNSF	NLITRTQSY	NLITRTQSY	NLITRTQSY	-	-	-
	G142D	Wuhan Hu-1	-	-	FCNDPFLGVY	DPFLGVYY	-	-	-
		BA.2	-	-	FCNDPFLDVY	DPFLDVYY	-	-	-
	V213G	Wuhan Hu-1	TPINLVRDL	TPINLVRDL	LVRDLPQGF	TPINLVRDL	-	-	-
		BA.2	TPINLGRDL	-	LGRDLPQGF	TPINLGRDL	-	-	-
	G339D	Wuhan Hu-1	-	-	NLCPFGEVF	NLCPFGEVF	GEVFNATRF	GEVFNATRF	GEVFNATRF
		BA.2	-	-	NLCPFDEVF	NLCPFDEVF	DEVFNATRF	DEVFNATRF	DEVFNATRF
	S371F/S373P/S375F/T376A	Wuhan Hu-1	-	-	ASFSTFKCY	YNSASFSTF	-	-	ASFSTFKCY
		BA.2	APFFAFKCY	-	VLYNFAPFF	APFFAFKCY	-	-	-
	D405N/R408S	Wuhan Hu-1	-	-	RQIAPGQTGKI	-	RQIAPGQTGKI	-	-
		BA.2	-	SFVIRGNEV	SQIAPGQTGNI	-	-	-	-
	K417N	Wuhan Hu-1	APGQTGKIA	-	GQTGKIADY	-	RQIAPGQTGKI	-	-
		BA.2	APGQTGNIA	-	GQTGNIADY	APGQTGNIADY	-	-	-
	N440K	Wuhan Hu-1	-	-	-	-	-	-	-
		BA.2	-	-	KLDSKVGGNY	-	-	-	-
Spike	S477N/T478K/E484A	Wuhan Hu-1	TPCNGVEGF	-	-	TPCNGVEGF	-	-	-
		BA.2	KPCNGVAGF	-	GVAGFNCYF	KPCNGVAGF	-	-	-
	Q493R/Q498R/N501Y/Y505H	Wuhan Hu-1	-	YQPYRVVVL	FQPTNGVGY	NCYFPLQSY	YQPYRVVVL	-	-
		BA.2	RPTYGVGHQP	HQPYRVVVL	RSYGFRPTY	FPLRSYGF	HQPYRVVVL	-	-
	D614G	Wuhan Hu-1	-	-	-	-	-	-	-
		BA.2	-	-	-	-	-	-	-
	H655Y	Wuhan Hu-1	-	-	IGAEHVNNSY	GAEHVNNSY	AEHVNNSY	AEHVNNSY	AEHVNNSY
		BA.2	-	-	GAEYVNNSY	GAEYVNNSY	AEYVNNSY	AEYVNNSY	AEYVNNSY
	N679K/P681H	Wuhan Hu-1	SPRRARSVA	SPRRARSV	-	-	-	-	-
		BA.2	-	KSHRRARSV	-	-	-	-	-
	N764K	Wuhan Hu-1	-	-	-	-	-	-	-
		BA.2	-	FCTQLKRAL	-	-	-	-	-
	D796Y	Wuhan Hu-1	-	-	-	TPPIKDFGGF	-	-	-
		BA.2	-	-	KQIYKTPPIKY	-	-	KQIYKTPPIKY	KQIYKTPPIKY
	Q954H	Wuhan Hu-1	VVNQNAQAL	VVNQNAQAL	VVNQNAQAL	VVNQNAQAL	-	-	-
		BA.2	VVNHNAQAL	VVNHNAQAL	-	-	-	-	-
	N969K	Wuhan Hu-1	-	LVKQLSSNF	LVKQLSSNF	LVKQLSSNF	-	-	-
		BA.2	-	QLSSKFGAI	LVKQLSSKF	LVKQLSSKF	-	-	-

Table S16. *In silico* predictions of BA.2 variant peptide binding to common UK HLA-C alleles. NetMHCpan epitope predictions for binding of BA.2 variant mutated regions to common UK HLA alleles. Weak binding is annotated blue, strong binding is annotated yellow and the HLA binding core in each instance is shown with the mutated amino acid(s) marked in red.

			NetMHCpan predicted HLA binding core with BA.2 mutation indicated						
	Mutation	Sequence	C03:03	C03:04	C04:01	C05:01	C06:02	C07:01	C07:02
Spike	T19I/24-26del/A27S	Wuhan Hu-1	LPPAYTNSF	LPPAYTNSF	LPPAYTNSF	-	TRTQLPPAY	TRTQLPPAY	TRTQLPPAY
		BA.2	RTQSYTNSF	RTQSYTNSF	-	RTQSYTNSF	NLITRTQSY	NLITRTQSY	NLITRTQSY
	G142D	Wuhan Hu-1	FCNDPFLGV	FCNDPFLGV	FCNDPFLGV	FCNDPFLGV	-	-	-
		BA.2	FCNDPFLDV	FCNDPFLDV	-	FCNDPFLDV	-	-	-
	V213G	Wuhan Hu-1	LVRDLPQGF	LVRDLPQGF	VRDLPQGF	-	SKHTPINLV	VRDLPQGF	VRDLPQGF
		BA.2	-	-	-	-	-	GRDLPQGF	-
	G339D	Wuhan Hu-1	-	-	-	-	-	-	-
		BA.2	-	-	PFDEVFNATRF	-	-	-	-
	S371F/S373P/S375F/T376A	Wuhan Hu-1	YNSASFSTF	YNSASFSTF	YNSASFSTF	-	YNSASFSTF	YNSASFSTF	YNSASFSTF
		BA.2	YNFAPFFAF	YNFAPFFAF	NFAPFFAF	VADYSVLYNF	VLYNFAPFF	YNFAPFFAF	YNFAPFFAF
	D405N/R408S	Wuhan Hu-1	-	-	-	RGDEVRQI	IRGDEVRQI	IRGDEVRQI	IRGDEVRQI
		BA.2	FVIRGNEV	FVIRGNEV	-	-	IRGNEVSQI	IRGNEVSQI	IRGNEVSQI
	K417N	Wuhan Hu-1	KIADYNYKL	KIADYNYKL	KIADYNYKL	KIADYNYKL	KIADYNYKL	KIADYNYKL	KIADYNYKL
		BA.2	NIADYNYKL	NIADYNYKL	NIADYNYKL	NIADYNYKL	NIADYNYKL	NIADYNYKL	NIADYNYKL
	N440K	Wuhan Hu-1	IAWNSNNL	IAWNSNNL	-	-	-	-	-
		BA.2	IAWNSNKL	IAWNSNKL	-	VIAWNSNKL	-	-	-
	S477N/T478K/E484A	Wuhan Hu-1	-	-	-	GVEGFNCYF	-	-	-
		BA.2	-	-	-	-	-	-	-
	Q493R/Q498R/N501Y/Y505H	Wuhan Hu-1	YQPYRVVVL	YQPYRVVVL	YFPLQSYGF	YQPYRVVVL	YQPYRVVVL	YQPYRVVVL	YQPYRVVVL
		BA.2	HQPYRVVVL	HQPYRVVVL	YFPLRSYGF	HQPYRVVVL	HQPYRVVVL	RSYGFRPTY	YFPLRSYGF
	D614G	Wuhan Hu-1	-	-	YQDVNCTEV	YQDVNCTEV	-	-	-
		BA.2	-	-	-	-	-	-	-
	H655Y	Wuhan Hu-1	-	-	-	-	-	-	-
		BA.2	-	-	-	-	-	TRAGCLIGAEY	TRAGCLIGAEY
	N679K/P681H	Wuhan Hu-1	-	-	-	-	NSPRRARSV	-	-
		BA.2	-	-	-	-	KSHRRARSV	-	SYQTQTKSH
	N764K	Wuhan Hu-1	FCTQLNRAL	FCTQLNRAL	-	-	NRALTGIAV	NRALTGIAV	NRALTGIAV
		BA.2	FCTQLKRAL	FCTQLKRAL	-	-	KRALTGIAV	KRALTGIAV	KRALTGIAV
	D796Y	Wuhan Hu-1	-	-	IKDFGGFNF	IKDFGGFNF	YKTPPIKDF	YKTPPIKDF	YKTPPIKDF
		BA.2	YKTPPIKYF	YKTPPIKYF	IYKTPPIKY	-	YKTPPIKYF	YKTPPIKYF	IYKTPPIKY
	Q954H	Wuhan Hu-1	VVNQNAQAL	VVNQNAQAL	VVNQNAQAL	VVNQNAQAL	VVNQNAQAL	-	-
		BA.2	VVNHNAQAL	VVNHNAQAL	VVNHNAQAL	VVNHNAQAL	VVNHNAQAL	-	
	N969K	Wuhan Hu-1	-	-	NFGAISSVL	-	SNFGAISSV	SNFGAISSV	-
		BA.2	-	-	KFGAISSVL	-	SKFGAISSV	SKFGAISSV	SKFGAISSV

UK COVIDsortium investigators

The members of the UK COVIDsortium investigators are Hakam Abbass, Aderonke Abiodun, Mashael Alfarih, Zoe Alldis, Daniel M Altmann, Oliver E Amin, Mervyn Andiapen, Jessica Artico, João B Augusto, Georgina L Baca, Sasha N L. Bailey, Anish N Bhuva, Alex Boulter, Ruth Bowles, Rosemary J Boyton, Olivia V Bracken, Ben O'Brien, Tim Brooks, Natalie Bullock, David K Butler, Gabriella Captur, Olivia Carr, Nicola Champion, Carmen Chan, Aneesh Chandran, Tom Coleman, Jorge Couto de Sousa, Xose Couto-Parada, Eleanor Cross, Teresa Cutino-Moguel, Silvia D'Arcangelo, Rhodri H Davies, Brooke Douglas, Cecilia Di Genova, Keenan Dieobi-Anene, Mariana O Diniz, Anaya Ellis, Karen Feehan, Malcolm Finlay, Marianna Fontana, Nasim Forooghi, Sasha Francis, Joseph M Gibbons, David Gillespie, Derek Gilroy, Matt Hamblin, Gabrielle Harker, Georgia Hemingway, Jacqueline Hewson, Wendy Heywood, Lauren M Hickling, Bethany Hicks, Aroon D Hingorani, Lee Howes, Ivie Itua, Victor Jardim, Wing-Yiu Jason Lee, Melaniepetra Jensen, Jessica Jones, Meleri Jones, George Joy, Vikas Kapil, Caoimhe Kelly, Hibba Kurdi, Jonathan Lambourne, Kai-Min Lin, Siyi Liu, Aaron Lloyd, Sarah Louth, Mala K Maini, Vineela Mandadapu, Charlotte Manisty, Áine McKnight, Katia Menacho, Celina Mfuko, Kevin Mills, Sebastian Millward, Oliver Mitchelmore, Christopher Moon, James Moon, Diana Muñoz Sandoval, Sam M Murray, Mahdad Noursadeghi, Ashley Otter, Corinna Pade, Susana Palma, Ruth Parker, Kush Patel, Mihaela Pawarova, Steffen E Petersen, Brian Piniera, Franziska P Pieper, Lisa Rannigan, Alicja Rapala, Catherine J Reynolds, Amy Richards, Matthew Robathan, Joshua Rosenheim, Cathy Rowe, Matthew Royds, Jane Sackville West, Genine Sambile, Nathalie M. Schmidt, Hannah Selman, Amanda Semper, Andreas Seraphim, Mihaela Simion, Angelique Smit, Michelle Sugimoto, Leo Swadling, Stephen Taylor, Nigel Temperton, Stephen Thomas, George D Thornton, Thomas A Treibel, Art Tucker, Ann Varghese, Jessry Veerapen, Mohit Vijayakumar, Tim Warner, Sophie Welch, Hannah White, Theresa Wodehouse, Lucinda Wynne, and Dan Zahedi

UK COVIDsortium Immune Correlates Network

The members of the COVIDsortium immune correlates network are Daniel M Altmann, Rosemary J Boyton, Tim Brooks, Benjamin Chain, Mala K Maini, Charlotte Manisty, Áine McKnight, James C Moon, Mahdad Noursadeghi, Thomas A Treibel
