# Supplementary material for: Immune boosting by B.1.1.529 (Omicron) depends on previous SARS-CoV-2 exposure
Source: Science. 2022 Jun 14:eabq1841. doi: 10.1126/science.abq1841 (PMC9210451; doi:10.1126/science.abq1841)
Supplement: Supplementary file 3 — Table S17 [file science.abq1841_table_s17.zip › science.abq1841_table_s17/science.abq1841_table_s17_caption.docx]

**Table S17.** abq1841. Raw data file (.xlsx)
